# Large studies reveal how reference bias limits policy applications of self-report measures

**DOI:** 10.1038/s41598-022-23373-9

**Published:** 2022-11-10

**Authors:** Benjamin Lira, Joseph M. O’Brien, Pablo A. Peña, Brian M. Galla, Sidney D’Mello, David S. Yeager, Amy Defnet, Tim Kautz, Kate Munkacsy, Angela L. Duckworth

**Affiliations:** 1 University of Pennsylvania, Philadelphia, USA; 2 University of Texas at Austin, Austin, USA; 3 University of Chicago, Chicago, USA; 4 University of Pittsburgh, Pittsburgh, USA; 5 University of Colorado-Boulder, Boulder, USA; 6 Mathematica, Inc., Princeton, USA

**Keywords:** Psychology, Human behaviour

## Abstract

There is growing policy interest in identifying contexts that cultivate self-regulation. Doing so often entails comparing groups of individuals (e.g., from different schools). We show that self-report questionnaires—the most prevalent modality for assessing self-regulation—are prone to *reference bias*, defined as systematic error arising from differences in the implicit standards by which individuals evaluate behavior. In three studies, adolescents (*N* = 229,685) whose peers performed better academically rated themselves lower in self-regulation and held higher standards for self-regulation. This effect was not observed for task measures of self-regulation and led to paradoxical predictions of college persistence 6 years later. These findings suggest that standards for self-regulation vary by social group, limiting the policy applications of self-report questionnaires.

## Introduction

Self-regulation refers to a diverse set of personal qualities, distinct from cognitive ability, that enable individuals to set and pursue goals. The terminology favored for self-regulation and its facets varies across the literatures of child development (e.g., effortful control, ego strength)^1–3^, adult personality (e.g., Big Five conscientiousness)^4^, psychopathology (e.g., impulse control)^5^, and economics (e.g., temporal discounting)^6,7^. Such diverse traditions in behavioral science have directed this attention because individual differences in self-regulation predict later life outcomes, including academic performance^8–10^; physical and mental health^11–13^; well-being and life satisfaction^14^; civic and social behavior^12,15^; job performance^16^; earnings^12,17–19^; and wealth^12,17^. Moreover, the effects of self-regulation are independent of, and comparable in magnitude to, cognitive ability and family socioeconomic status (SES)^8,12^.

A half-century of basic research suggests that self-regulation develops optimally in caring environments that encourage adaptive goal-relevant knowledge (e.g., strategies for managing attention), beliefs (e.g., that emotion and motivation can be regulated), and values (e.g., that self-regulation is important)^20^. This development extends far beyond early childhood, when children are mostly in the company and care of parents. Indeed, adolescence may be particularly important for supporting self-regulation because of the rapid growth, learning, adaptation, and neurobiological development that mark this period of life^21–23^. Further, impulsive choices in adolescence (e.g., to start smoking, to drop out of school) can alter life trajectories in ways that are difficult to reverse^12^.

Schools are a natural target for policy because of their potential to provide equal access to environments that support the development of self-regulation^24,25^. Not only is school where young people spend most of their waking hours outside the home, it is also where they experience a multitude of factors that have been shown to either scaffold or stymie the development of self-regulation, including adult role models^26,27^ and peers^28,29^. Recently, a growing chorus of policymakers has urged schools to extend their purview beyond traditional academic coursework and into the domain of social-emotional skills such as self-regulation—a trend that is reflected in the expanded scope of federal and state standards and accountability systems^30–32^.

In this investigation, we identify a pervasive measurement bias that, if not remedied, may thwart policymakers’ efforts to evaluate, measure, and improve the effectiveness of schools that foster adolescent self-regulation. The possibility of this measurement bias has led to serious questions from policymakers about “whether we can make [self-regulation skills] visible, comparable, and therefore amenable to deliberate policy action in a similar way that traditional tests do with academic knowledge and skills”^33^. As a result, education systems have been left with great interest in self-regulation and related constructs—but insufficient scientific guidance.

The empirical starting point for our research is the mixed and often counterintuitive evidence regarding school effects on self-regulation. On one hand, Jackson et al.^34^ show encouraging evidence that schools can differ in how much they improve students’ scores on a self-report measure of hard work, and these school differences predicted students’ later college enrollment and persistence. On the other hand, evaluations of charter schools show that they fail to raise self-reports of self-regulation, despite raising report card grades, standardized test scores, attendance rates, and college enrollment levels while reducing incarceration and unplanned pregnancies^35–38^. Are high-performing schools whose cultures explicitly emphasize hard work and high expectations^39,40^ in fact having no impact on students’ self-regulation—or is there a problem in how self-regulation is measured?

**Figure 1:**
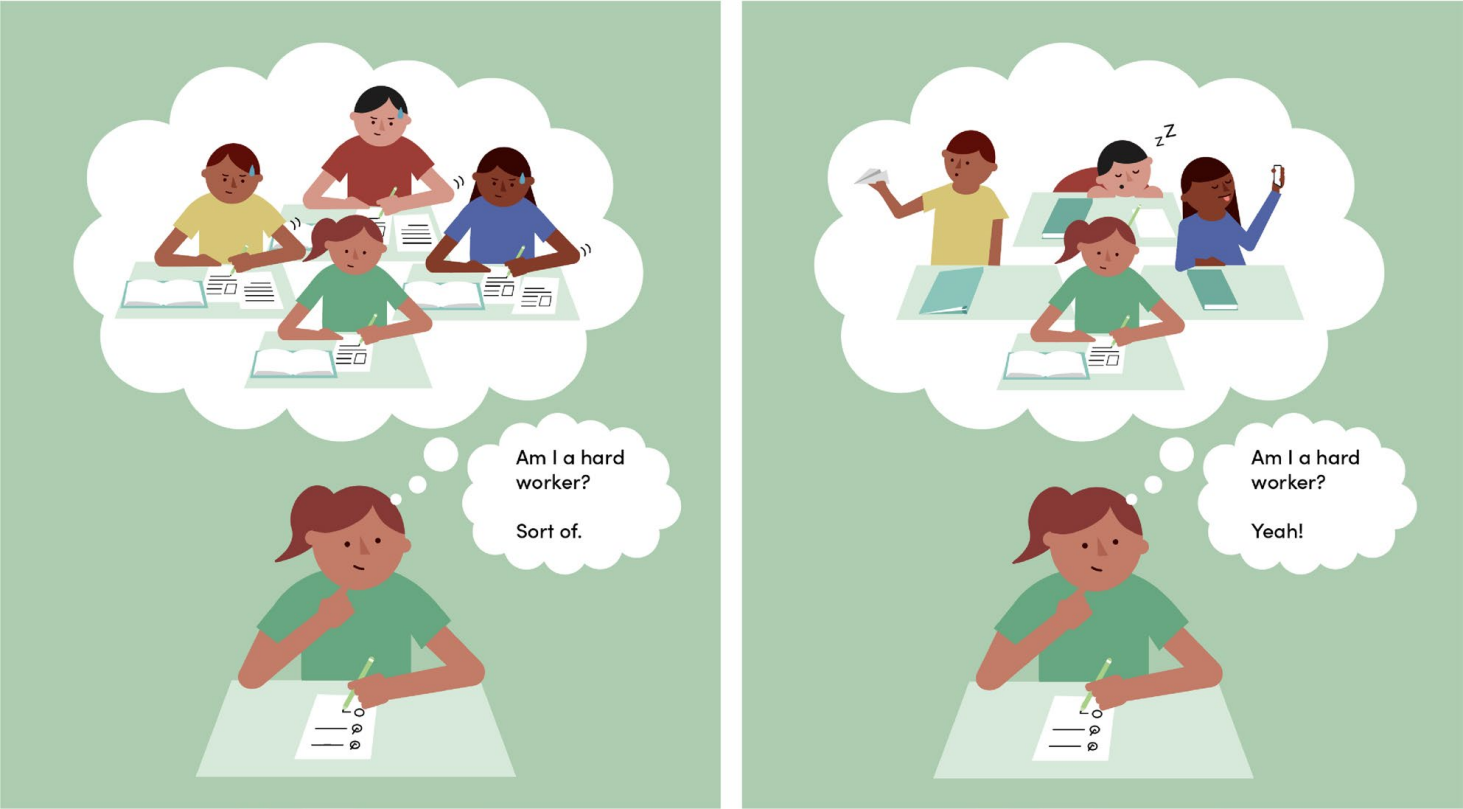
Peers influence the standards by which an individual judges their own behavior, resulting in a “reference bias” effect that distorts cross-context comparisons of self-reported self-regulation. Illustration by Tom McQuaid.

We suggest that reference bias, the systematic error that arises when respondents refer to different implicit standards when answering the same questions^41^, is a legitimate threat to between-school comparisons and can help explain the conflicting evidence of school effects on self-regulation. Moreover, we contend that even within a school, comparisons of students are biased when different subgroups of students rely on different standards when answering the same questions. In the present policy context, reference bias is especially pernicious because it is difficult to detect and diagnose. Unlike social desirability bias, modesty bias^42^, faking, and response style biases^43^, reference bias can emerge even when respondents answer truthfully, and it can coexist with otherwise strong validity associations at the individual level. This is because reference bias can distort inferences any time there are comparisons of self-regulation across *groups* who differ in their frames of references—for example, schools with very different peer cultures with respect to effort, or even subcultures within a school.

Why might self-report questionnaires be subject to reference bias? Dominant models in survey methodology identify a multi-stage cognitive response process: students first read and interpret the question; then they identify relevant information in memory, form a summary judgment, and translate this judgment into one of the response options; finally, they edit their response if motivated to do so^44–46^. As illustrated in Fig. 1, a student may interpret a questionnaire item and its response options differently depending on their peers’ typical behaviors^47^. If they have high-achieving classmates who, for example, study for hours each evening and consistently arrive prepared for class, they might judge themselves against higher standards and rate themselves lower in self-regulation than an equally industrious student whose lower-achieving peers study and prepare less. While schools might be effective in increasing self-regulated behavior, they might at the same time increase the standards, leading to lower self-reported self-regulation.

A well-established research literature has demonstrated that the subjective view students hold of themselves, both in general terms (i.e., self-esteem) and in the realm of academic performance (i.e., academic self-concept) depends upon peer comparisons^42,48,49^. In particular, the Big Fish Little Pond Effect (BFLPE) refers to the lower academic self-concept of students in higher-achieving schools^50^. A related and older literature on social comparison has demonstrated that in general, people spontaneously compare themselves to other people, especially to people who are superior to them in some way, which can lower their subjective appraisal of their own ability^51^. Finally, there is evidence that academic self-concept and standardized test scores are positively correlated within countries but inversely correlated between countries—a phenomenon dubbed the attitude-achievement paradox^42,52^. In sum, there is ample evidence for the influence of peers on inherently subjective constructs.

In contrast, evidence that reference bias distorts comparisons of self-regulation across social groups has been indirect. A handful of cross-cultural studies have yielded paradoxical findings (e.g., Asian countries such as Japan and South Korea ranking lower in self-reported conscientiousness than other countries that are typically thought to be less conscientious^53^), but none of these studies directly measured standards for behavior, relying instead on experts’ ratings of cultural stereotypes or indirect proxies for self-regulation (e.g., the average walking speed in downtown locations of a convenience sample of a country’s residents, as a proxy for the nation’s conscientiousness).

In the educational literature, studies that compare the test scores and average self-regulation scores for different schools have not ruled out unobserved confounds, such as the possibility that school factors (e.g., average family income) that increase test scores (e.g., due to investment in educational opportunities) also decrease self-regulation (e.g., by shielding children from responsibilities that could cultivate self-regulation). Therefore, the research literature to date has not been able to distinguish biases in self-reports from potentially true group differences in self-regulation.

In this investigation, we overcome these limitations by using three complementary methods to examine reference bias more directly than has been possible previously. Our approach is motivated by the basic finding that people judge themselves compared to salient and similar others^47^. Therefore we exploit (Studies 1 and 2) or work around (Study 3) variation in people’s reference groups.

In Study 1 (total *N* = 206,589 students in *k* = 562 Mexican high schools), we show that the reference bias effect appears even within the same school in a year-over-year comparison. When students are surrounded by higher-achieving peers relative to other students at the same school in a different year, they rate themselves lower in self-regulation. Study 2 addresses an additional confound that could remain in Study 1’s analysis, which is the possibility that year-over-year fluctuations in test scores are not random but are due to choices made by families about the academic trajectory of the school. In Study 2 (*N* = 21,818 students in *k* = 62 U.S. secondary schools), we rule this out with an analysis rooted in the purported psychological explanation for reference bias, which is that people’s self-judgments should be more influenced by the peers whose behaviors they observe rather than peers whose behaviors they do not observe. We show that reference bias is evident in data from a single school year only when administrative data showed that the peers shared classes and therefore had an opportunity to observe each other’s self-regulated behavior. Furthermore, Study 2 examined the theorized, but typically unmeasured, explanation for reference bias: differences in students’ implicit standards for self-regulation (i.e., how many hours of homework constitute “a lot of homework” and how often it means to “sometimes” forget what they need for class).

Studies 1 and 2 argue against school-level alternative explanations for reference bias but nevertheless allowed for the possibility that high-achieving peers reduce a student’s real capacity for self-regulation. Study 3 (*N* = 1278 seniors in *k* = 15 U.S. high schools) addressed this possibility with a workaround: an objective behavioral task that involves no self-reports and therefore is not subject to biases due to differences in frames of reference. By matching self-regulation data collected in high school to records of college graduation, we show that there is no evidence of reference bias when a behavioral task is used. This evidence is bolstered by Study 3’s use of a measure of school achievement that is independent of the high school peer group: graduation from college within 6 years after high school completion.

## Study 1: Evidence for reference bias in a country-wide natural experiment

In 2012 and 2013, the Secretariat of Public Education administered questionnaires measuring grit (the passion and perseverance for long term-term goals^54^) and collected data on academic performance from high school seniors in a nationally representative sample of 10% of high schools in Mexico. We analyzed data from the 1% of all schools that, by chance, were selected in both years. This enabled us to exploit exogenous variation in the academic performance of the 2013 high school cohort when compared to the performance of the 2012 cohort. Reference bias was quantified as the effect on self-reported grit uniquely attributable to peer academic performance (i.e., the cohort-wide averages of GPA, standardized math test scores, and standardized reading test scores, respectively, excluding said student from the average), after controlling for differences between schools, cohort year, and each student’s own academic performance.

### Methods

#### Sample and procedure

High school seniors in two representative random samples, each comprising 10% of schools in Mexico, completed standardized achievement tests of math and reading and, separately, self-report questionnaires late in the spring term of the 2011–2012 and 2012–2013 academic years, respectively. By chance, about 1% (*k* = 562) of high schools were included in both years. Our final sample includes 97.8% of the students in these high schools (*N* = 206,589) who completed a questionnaire measure of grit. There were slightly more girls than boys in our sample (53.49% female). On average, students in our sample were 17.61 years old (SD = 0.79).

#### Self-reported grit

The Technical Committee for Background Questionnaires at the National Center of Evaluation for Higher Education in Mexico (Centro Nacional de Evaluación para la Educación Superior) translated all 8 items of the Short Grit Scale^55^ as well as its 5-point Likert-type response scale (1 = *Not at all like me* to 5 = *Very much like me*) into Spanish. The observed reliability was $\alpha = 0.62$. All reported reliabilities are Cronbach’s alphas.

#### Grade point average (GPA)

Students reported their overall, verbal, and math GPAs using a categorical scale which ranged from *less than 5.9* to *10* in half-point increments (i.e., < 5.9, 6.0–6.4, 6.5–6.9, etc.). We used the midpoint of the range in our analyses (i.e., 5.7, 6.2, 6.7, etc.). Although official GPAs were not available, meta-analytic estimates of the correlation between self-reported and objectively recorded GPA is $r =0.82$^56^. To avoid any issues with multicollinearity, we ran separate models for each GPA measure.

#### Standardized test scores

The Mexican Secretariat of Public Education provided standardized math and reading scores.

#### Analytic strategy

We used ordinary least squares (OLS) regression with clustered standard errors to predict self-reported grit from student’s own and peer’s academic performance:

$$\begin{aligned} G_{ist}=\alpha a_{ist}+\gamma b_{-ist}+ \theta _s+ \eta _t+\varepsilon _i \end{aligned}$$

where $G_{ist}$ is the self-reported grit for student *i* who was in 12th grade in school *s* at time *t* (2012 or 2013). Term $a_{ist}$ is that student’s own academic performance, operationalized as self-reported GPA, standardized math scores, or standardized verbal scores, respectively. Term $b_{-ist}$ represents the average academic performance of students sharing a school with each student *i*, excluding student *i*. Term $\theta _s$ represents fixed effects for each student’s school and captures ways in which schools might differ from each other—including such differences as teachers, curricula, school policies, and regional populations from which schools draw their members. Term $\eta _{t}$ (fixed effect for year), captures how cohorts for each school systematically differ from each other. $\epsilon _i$ represents error.

### Results

#### Students surrounded by higher-performing classmates rate themselves lower in grit

**Figure 2:**
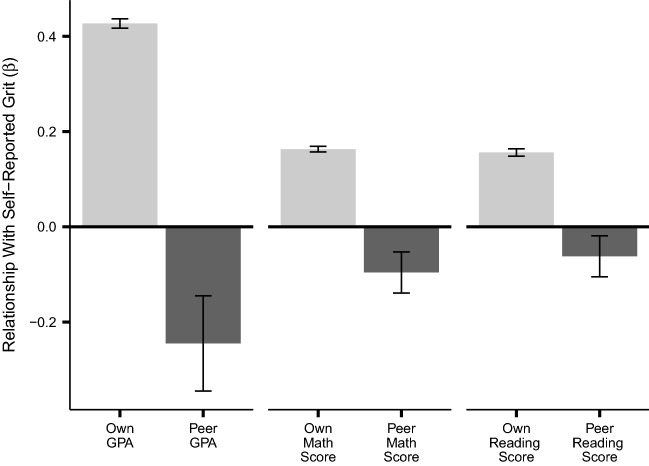
In Study 1, self-reported grit correlated positively with a student’s own academic performance but inversely with the performance of their schoolmates. OLS models included demographic controls and school fixed effects. Error bars represent 95% confidence intervals. Model $R^2$s for GPA, math score, and language score were 0.124, 0.071, and 0.071, respectively.

Consistent with prior research, among students in the same school, self-reported grit correlated positively with GPA ($\beta$ = 0.43, *p* < 0.001), standardized math test scores ($\beta$ = 0.16, *p* < 0.001), and standardized reading test scores ($\beta$ = 0.16, *p* < 0.001). However, consistent with reference bias, self-reported grit correlated inversely with schoolmates’ GPA ($\beta = -0.25$, *p* < 0.001), peer standardized math test scores ($\beta = -0.09$, *p* $< 0.001$), and peer standardized reading test scores ($\beta = -0.07$, $p = 0.004$). See Fig. 2 and Supporting Information for details.

#### Evidence for reference bias was consistent across demographic subgroups

Capitalizing on the size and representativeness of our sample, we explored moderators of reference bias. Regression coefficients for peer academic performance were not significantly different across subgroups defined by gender, mother’s educational level, school type (public or private), or school size. See Tables S6 and S7 in Supporting Information for details.

## Study 2: Replication and extension in a single large school district

In Study 2, we partnered with the nonprofit organization Character Lab to replicate and extend Study 1 with a sample of students in grades 8 through 12 in a large, diverse school district in the United States. This partnership enabled us to obtain official class schedules for each student, which we used to distinguish near- versus far-peers as students who did or didn’t share daily academic classes, respectively. Whereas GPA was self-reported in Study 1, in Study 2 we obtained GPA from official school records. As part of a larger survey administered by Character Lab, students completed a self-report questionnaire of conscientiousness (the tendency to be organized, responsible, and hardworking^57^) as well as two questions we developed to directly assess self-regulation standards.

### Methods

#### Sample and procedure

This study included data from *N* = 21,818 (50% female, $M_{age}$ = 15.60, $SD_{age}$ = 1.54) students attending *k* = 62 middle and high schools in a large public school district in the United States who completed surveys in either October 2019 or February 2020. This district was part of Character Lab Research Network (CLRN), a consortium of school partners committed to advancing scientific insights that help children thrive. According to school records, the race/ethnicity of our sample was: Hispanic/Latinx (41%), White (28%), Black (23%), and other (8%). About half (49%) of students were eligible for free and reduced-price meals.

#### Self-reported conscientiousness

Students completed 12 items from the Big Five Inventory-2^58^ assessing conscientiousness (e.g., “I am someone who is persistent, works until the task is finished”) using a 5-point Likert-type scale ranging from 1 = *Not like me at all* to 5 = *Totally like me*. The observed reliability was $\alpha = 0.83$.

#### Standards for hard work and preparedness

We included two questions to measure implicit standards for self-regulation. One question assessed norms for hard work: “If a student in your grade says they did ‘a lot of homework’ on a weeknight, how long would you guess they mean?” Eight response options ranged from 15 min (coded as 0.25 hours) to 3 or more hours (coded as 3 hours). The second question assessed norms for preparedness: “If a student in your grade says they ‘sometimes’ forget something they need for class, how often would you guess they mean?” Seven response options ranged from *once a month* to *three times or more per day* (coded as 66 times per month). We reverse-coded these values such that higher numbers indicated stricter standards for preparedness. These items were created for this study and used here for the first time.

#### Grade point average (GPA)

From school administrative records, we calculated GPAs on a 100-point scale by averaging final grades in students’ academic courses (English language arts, math, science, social studies) for the quarter in which students took the survey during the 2019–2020 school year.

#### Near-peer and far-peer GPAs

For each student, we designated near-peers as those students who took at least one academic course with the target student during the quarter in which they took the survey. We designated far-peers as students in the same school who did *not* share any academic courses. For the average student in our sample, 38% of schoolmates were near-peers and 62% were far-peers.

#### Analytic strategy

To examine whether self-regulation standards and conscientiousness related to students’ own and peers’ performance, we fit OLS regression models with standard errors clustered by school to estimate the following equation:

$$\begin{aligned} S_{is} = \alpha a_{is}+ \gamma _{1}b_{-is}+\gamma _{2}c_{-is}+\delta x_{is}+ \theta _s+\varepsilon _i, \end{aligned}$$

where $S_{is}$ is a survey measure of conscientiousness or self-regulation standards for student *i* in school *s*, $a_{is}$ is a student’s own GPA, $b_{-is}$ is the average GPA of students in the same school sharing at least one academic course with student *i*, $c_{-is}$ is the average GPA of students in the same school but not sharing any academic courses with student *i*, $x_{is}$ is a vector of student characteristics (age, gender, race/ethnicity, grade level, free or reduced-price meal status, English language learner status, special-education status, home language, and timing of the survey), $\theta _{s}$ represents school fixed effects, and $\epsilon _i$ is is a random error term.

### Results

#### Reference bias replicates: students whose classmates perform better academically rate themselves as lower in conscientiousness. As expected, this effect is driven by near-peers rather than far-peers

**Figure 3:**
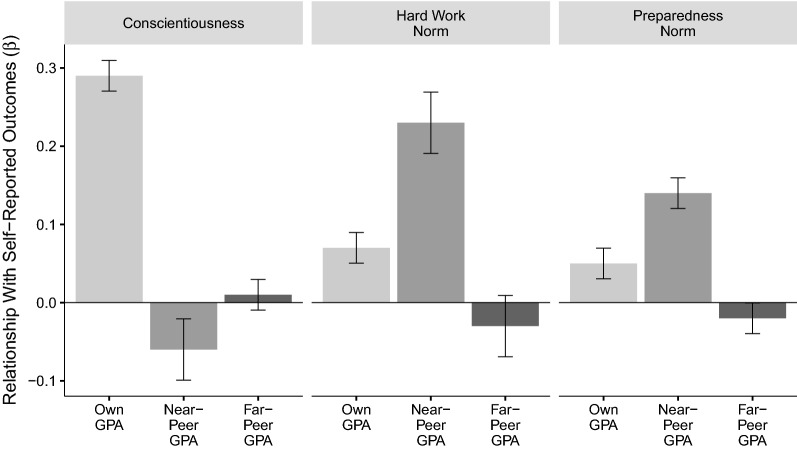
In Study 2, self-reported conscientiousness correlated positively with a student’s own GPA and negatively with the GPA of near-peers. In contrast, standards for what constitutes hard work and preparedness correlated positively with both own and near-peer GPA. As expected, there was no effect of far-peer GPA. OLS models included demographic controls and school fixed effects. Error bars represent 95% confidence intervals. Model $R^2$s for conscientiousness, hard work norms, and preparedness norms were 0.095, 0.159, and 0.059, respectively.

If implicit standards for self-regulation are determined by social comparison, reference bias should be driven by the individuals with whom individuals are in direct contact. As shown in Fig. 3, consistent with Study 1, self-reported conscientiousness was correlated positively with a student’s own GPA ($\beta = 0.29$, $p< 0.001$), negatively with the GPA of near-peers ($\beta = - 0.06$, $p < 0.001$), and not at all with the GPA of far-peers ($\beta = 0.01$, $p = 0.395$). See Table S10 in Supporting Information for details.

#### Students whose near-peers perform better academically hold higher self-regulation standards

As expected, standards for hard work were predicted by a student’s own GPA ($\beta = 0.07$, $p < 0.001$) and the GPA of their near-peers ($\beta = 0.23$, $p < 0.001$), but not the GPA of their far-peers ($\beta = -0.03$, $p = 0.198$). The same pattern emerged for preparedness norms, which were predicted by students own GPA ($\beta = 0.05$, $p < 0.001$) and the GPA of their near-peers ($\beta = 0.14$, $p < 0.001$), but not far-peers ($\beta = -0.02$, $p = 0.080$). As in Study 1, the patterns of findings were generally similar across subgroups. See Tables S11–S16 in Supporting Information for details.

## Study 3: In a longitudinal study of college graduation, evidence of reference bias in questionnaire but not in task measures of self-regulation

In Study 3, we sought evidence of discriminant validity. Unlike questionnaires, which require participants to make subjective judgments of their behavior, task measures assay behavior directly. In a prospective, longitudinal study of *N* = 1278 students attending *k* = 15 different college-preparatory charter schools in the United States, we tested the prediction that reference bias should be evident in questionnaire but not behavioral task measures of self-regulation. In their senior year of high school, students self-reported their grit and self-control (the ability to be in command of one’s behavior and to inhibit one’s impulses^57^). In addition, they completed the Academic Diligence Task, a behavioral task in which students voluntarily allocate attention to either good-for-me-later math problems or fun-for-me-now games and videos. The Academic Diligence Task has previously been validated as indexing self-control and grit^59,60^. Six years later, we used the National Student Clearinghouse database to identify students who successfully obtained their college diploma.

### Methods

#### Sample and procedure

A few weeks before graduation, *N* = 1278 (55% female, $M_{age}$ = 18.01, $SD_{age}$ = 1.01) high school seniors responded to self-report questionnaires and task measures in school computer labs. Students attended *k* = 15 charter schools located in various urban centers in the United States. Between 76 and 98% of the students at each school participated in the study. Most students were socioeconomically disadvantaged (84% of students’ mothers had less than a 4-year degree, 68% qualified for free or reduced-priced meals), and were mostly Latinx (46%) and African American (40%).

#### Self-reported grit

Students completed a 4-item version of the Grit Scale developed specifically for adolescents^61^. Students responded on a 5-point Likert-type scale ranging from 1 = *Not at all true* to 5 = *Completely true*. The observed reliability was $\alpha = 0.78$.

#### Self-control

Students completed four items from the Domain-Specific Impulsivity Scale^59,62^ assessing academic self-control (e.g., “I forgot something needed for school”). Students responded on a 5-point Likert-type scale ranging from *Not at all true* to *Completely true.* The observed reliability was $\alpha = 0.72$.

#### Academic Diligence Task (ADT)

A subset (*n* = 802) of students in our sample completed the Academic Diligence Task, a behavioral assessment of self-regulation that has been validated in separate research^59^. This computer-based task begins with screens explaining that practicing simple mathematical skills like subtraction can aid in further enhancing overall math abilities. Then, they completed three 3-min timed task blocks. In each, they chose between “Do math” and “Play game or watch movie.” Clicking “Do math” displayed a math task involving single-digit subtraction with multiple-choice responses. On the other hand, clicking “Play game or watch movie” allowed students to play Tetris or watch entertaining videos. Students could freely switch between them during each block. See Supporting Information for details. The key metric from the ADT was the mean number of problems correctly answered over the three blocks. Basic subtraction is very easy for most 12th grade students, so attentive engagement with the task resulted almost exclusively in correct answers: The median rate of correct responses was 98.3%. Due to positive skew and some clustering of data at 0 (i.e., students who did no math problems), we applied a square-root transformation to minimize bias from extremely high scores; this created an approximately normal distribution, which we used in subsequent calculations. Models using raw (i.e., non-transformed) ADT scores are shown in Table S19. Across the three blocks, the observed reliability was $\alpha = .78$.

#### General cognitive ability

During the online survey, students completed a brief (12-item) version of Raven’s Progressive Matrices as an assessment of general cognitive ability^63^. The ability variable was calculated as the sum of correctly answered questions out of 12, with any missing questions marked as incorrect. The observed reliability was $\alpha = 0.73$.

#### College graduation

We matched our data to the National Student Clearinghouse, a public database that includes enrollment and graduation data for over 97% of students in 2022^64,65^. We coded six-year college graduation as 1 = *obtained degree within 6 years of enrollment* and 0 = *did not obtain degree within 6 years of enrollment.*

#### Analytic strategy

Because we were interested in both individual-level and school-level differences in self-regulation, we used multilevel modeling to analyze how the Academic Diligence Task and self-reported grit and self-control, predict college graduation. Specifically, we expected the ADT to positively predict college graduation at both the within- and between-school levels. We expected the relationship to be positive because prior research shows that students who obtain higher ADT scores tend to perform better academically^59^. Moreover, we expected the relationship to be positive at *both levels* because, as a task measure, it does not involve comparative judgment and thus cannot be influenced by reference bias. In contrast, we expected self-reported grit and self-control to positively predict college graduation within a school but negatively between schools. We used a missing dummy variable coding approach to deal with missing data and included controls for general cognitive ability in our models.

### Results

#### Evidence of reference bias in longitudinal predictions of college graduation from self-reported, but not objectively measured, self-regulation

**Figure 4:**
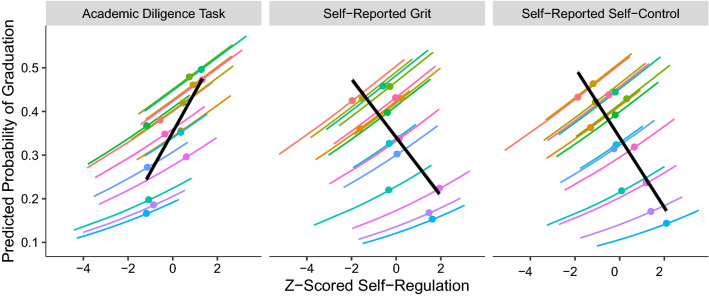
In Study 3, comparing students within schools (colored lines), higher self-regulation predicted higher odds of college graduation, whether measured by self-report questionnaires for grit and self-control or by a behavioral task called the Academic Diligence Task. When comparing schools to each other, however, higher self-reported grit and self-control scores predicted *lower* graduation rates, whereas the behavioral task *positively* predicted college graduation, as shown in the solid black lines. Plots show predicted probabilities of graduation from multilevel logistic regression models. *AUC*s for models predicting the academic diligence task, self-reported grit, and self-reported self-control were 0.694, 0.693, and, 0.676, respectively.

As shown in Fig.  4, among seniors in the same high school, higher scores on self-report questionnaires of self-control (*b* = 0.16, *OR* = 1.17, *p* = 0.022) and grit (*b* = 0.16, *OR* = 1.18, *p* = 0.020) each predicted greater odds of earning a college diploma 6 years later. However, college graduation rates were actually lower for schools with higher self-reported self-control and grit scores ($b = -0.44$, *OR* = 0.64, *p* = 0.001; $b = -0.39$, *OR* = 0.68, *p* = 0.005, for self-control and grit, respectively).

This paradoxical pattern was not evident when self-regulation was assessed objectively using the Academic Diligence Task^59^. Among seniors in the same school, college graduation was predicted by higher scores on the Academic Diligence Task (*b* = 0.15, *OR* = 1.17, *p* = 0.031). Likewise, when comparing across schools, college graduation rates were higher for schools whose students performed better on the Academic Diligence Task (*b* = 0.46, *OR* = 1.58, *p* < 0.001).

Taken as a whole these findings suggest that reference bias reversed the relationship between self-regulation and graduation across schools. See Supporting Information for summaries of multilevel logistic regression models, robustness checks, and a replication of the own versus peer performance models in Studies 1 and 2.

## Discussion

The three studies in this investigation provide direct evidence for reference bias in self-reported self-regulation. In Study 1, high school seniors rated themselves lower in grit when their schoolmates earned higher GPAs and standardized achievement test scores. In Study 2, we replicated this effect using self-report questionnaires of conscientiousness and showed that it was driven by near-peers rather than by far-peers. Further, we showed that the GPA of near-peers (but not far-peers) correlates positively with self-regulation standards. Finally, in Study 3, we found that using self-report questionnaires of grit and self-control to predict college graduation 6 years later produced paradoxical results: Within a high school, students with higher self-reported self-regulation were more likely to graduate from college 6 years later, but across schools, average levels of self-regulation negatively predicted graduation. In contrast, an objective task measure of self-regulation—which indexed performance directly and did not ask students to judge themselves—positively predicted college graduation both within and across schools.

How big are reference bias effects? Studies 1 and 2 provide estimates in the range of *r* = 0.06 to 0.25. All else being equal, a student in our samples whose peers’ academic achievement is one standard deviation above the mean is predicted to rate their own self-regulation as 10–20% of a standard deviation lower. Assuming that higher standards for self-regulation depress self-report ratings while at the same time, via social norms and modeling, encourage more self-regulated behavior, these are actually lower-bound estimates. Consistent with this possibility, when we use a behavioral task to assess self-regulation, we observe results consistent with positive peer effects (Study 3), which have also been previously reported in the literature^66–68^. Taken together, our findings suggest that reference bias effects, even across social groups in the same country, can be at least small-to-medium in size by contemporary benchmarks^69^ and comparable to the effect sizes for faking on self-regulation questionnaires in workplace settings^70^.

Several limitations of the current investigation suggest promising directions for future research.

First, we must be cautious about drawing strong causal inferences from the non-experimental data in our three field studies. In Study 1, variation in peer quality could have influenced self-reported self-regulation for reasons other than reference bias. Against this, we found direct evidence for near-peer influence on self-regulation standards provided in Study 2. However, in Study 2, there is the possibility of reverse-causality. For example, rather than near-peers determining self-regulation standards, it is possible that self-regulation standards determined patterns of enrollment (e.g., students with higher standards self-selecting into the same difficult classes). In Study 3, we cannot rule out the possibility that some unmeasured confound gave rise to contradictory within-school versus between-school results on self-report (but not objective task) measures of self-regulation. In sum, it is important to confirm our observational findings by experimentally manipulating peer groups and/or standards of self-regulation.

Second, there are limits to the external validity of our conclusions. In particular, we examined reference bias in adolescence, a developmental period in which sensitivity to peers is at its apogee^71^. The adolescents in our investigation lived in Mexico (Study 1) and the United States. (Studies 2 and 3). Further research on children and adults, in a wider sample of countries, and in contexts outside formal schooling, is needed to establish boundary conditions and moderators of reference bias. In general, effect sizes for reference bias are expected to be smaller when comparing social groups with more similar standards.

Third, we did not collect nuanced data on social networks (e.g., friendships, acquaintances). Indeed, our operationalization of peer groups was quite crude—students in the same grade and attending the same school in Study 1 and 3, and students in the same grade and school who share at least one academic class (i.e., near-peers) in Study 2. Given the increasing prevalence of social-network studies and the continued popularity of self-report questionnaires in behavioral science, it should be possible to identify the influence of prominent social referents and close friends on reference bias.

Finally, while we collected information about student’s standards for self-regulation (in Study 2) and an objective measure of self-regulation (in Study 3), we have yet to collect both types of measures in the same sample. Doing so in a future study would enable us to test a mediation model in which peers influence standards for self-regulation which, in turn, diminish self-reported self-regulation relative to performance on a behavioral task of self-regulation. More generally, additional research is needed to establish the mediators, moderators, and boundary conditions of reference bias in the measurement of self-regulation.

Unfortunately, the problem of reference bias is not easily corrected. The most commonly suggested solution is anchoring vignettes^72^. This technique entails asking participants to rate detailed descriptions of hypothetical characters. These ratings are then used to adjust self-report questionnaire scores upward or downward depending on the stringency or leniency with which participants evaluated the hypothetical characters. Anchoring vignettes can increase the reliability and validity of self-reports^73^ but do not always work as intended^74^. They also increase the time, effort, and literacy required from survey respondents, which may limit their utility at scale^73,75^.

A related possibility is to use behaviorally anchored^76^ or act-frequency rating scales^77^, which ask respondents to rate themselves on more specific, contextualized behaviors than is typical in traditional questionnaires. For example, while students at over-subscribed charter schools do not rate themselves as more self-regulated, they and their parents do report more “minutes of homework completed” in an open-ended question in the same questionnaire^38^. In our view, such questions might mitigate response bias but probably do not eliminate it altogether. Why not? Because all subjective judgments rely, at least to some degree, on implicit standards that can differ (e.g., What level of effort is sufficient to consider yourself to be “doing homework”?).

As shown in Study 3, self-regulation can be assessed with behavioral tasks, which appear immune to reference bias. However, task measures have their own limitations, including a dramatically lower signal-to-noise ratio when compared to questionnaires and, relatedly, surprisingly modest associations with other measures of self-regulation^46,78–81^.

Perhaps the best means of obviating reference bias is to take a multi-method, multi-informant approach to assessment, including trained observers who can rate behavior across multiple occasions^12^. Observers who have seen hundreds, if not thousands, of cases typically have a wider reference frame than the individuals they are evaluating, which might explain why teacher ratings of behavior are more reliable and predictive of future outcomes than either parental reports or student self-reports^82^. The rarity of multi-method and multi-informant approaches suggests that, unfortunately, few researchers have the necessary resources or expertise to implement it, particularly at scale.

What are the implications of reference bias for researchers and policymakers?

Reference bias could suppress, or even reverse, the measured effects of interventions if the standards by which people judge their own behavior on pre- and post-questionnaires shift as a function of the intervention^83^. In one study, participants were asked to rate their interviewing skills before training (*pre*). Afterward, participants rated themselves again (*post*) and, in addition, retrospectively estimated what their skills had been at baseline (*then*). Even though questionnaire items were identical for all assessments, *then* ratings were lower than *pre* ratings—suggesting that participants adopted higher standards as a result of the intervention. Moreover, third-party judges’ ratings of performance matched *then*-*post* change better than *pre*-*post* differences^84^.

The implications of reference bias extend beyond intervention research. Consider, for example, mean-level increases in conscientiousness from adolescence through midlife^85–87^. If adults in their 50s hold higher standards for what it means to be courteous, rule-abiding, and self-controlled than teenagers, then age differences in conscientiousness may be even larger than we now think. In fact, to the extent that implicit standards and actual behavior are inversely correlated, reference bias should be expected to attenuate associations of self-regulation with groups of any kind.

While the importance of personal qualities like self-regulation is incontrovertible, the specter of reference bias argues against relying on self-report questionnaires when comparing students attending different schools, citizens who live in different countries, or indeed any of the members of any social group whose standards could differ from one another. Are you a hard worker? Responding to such a question requires looking *within* to identify the patterns of our behavior. In addition, the evidence for reference bias presented here suggests that knowingly or not, we also look *around* when we decide how to respond.

### Ethics statement

All methods were carried out in accordance with relevant guidelines and regulations. Participants in Studies 2 and 3 completed written informed consent prior to participation in this study. Participants in Study 1 were completing country-mandated educational assessments, and thus did not complete written informed consent. We accessed this secondary dataset with authorization from the Mexican Secretariat of Education. Study 1 was approved by the Mexican Secretariat of Education. Study 2 was approved by Advarra IRB. Study 3 was approved by Stanford University IRB.

## Supplementary Information

Supplementary Information.

## Data Availability

The data that support the findings of Study 1 are available from the Mexican Ministry of Education but restrictions apply to the availability of these data, which were used under license for the current study, and so are not publicly available. Data are however available from the authors upon reasonable request and with permission of the Mexican Ministry of Education. Data for Study 2 and Study 3 are included in this published article’s Supporting Information.
