# Supplementary material for: Large studies reveal how reference bias limits policy applications of self-report measures
Source: Sci Rep. 2022 Nov 10;12:19189. doi: 10.1038/s41598-022-23373-9 (PMC9649615; doi:10.1038/s41598-022-23373-9)
Supplement: Supplementary file 1 — Supplementary Information. [file 41598_2022_23373_MOESM1_ESM.pdf]

# Supplementary Information for

## Large Studies Reveal How Reference Bias Limits Policy Applications of Self-Report Measures

**Benjamin Lira*, Joseph M. O'Brien*, Pablo A. Peña, Brian M. Galla, Sidney D'Mello, David S. Yeager, Amy Defnet, Tim Kautz, Kate Munkacsy, Angela L. Duckworth**

**Benjamin Lira.**
**E-mail: blira@upenn.edu**

### This PDF file includes:

Supplementary text
Figs. S1 to S3
Tables S1 to S21
Legends for Dataset S1 to S2
SI References

### Other supplementary materials for this manuscript include the following:

Datasets S1 to S2

## List of Figures


S1 Details about the construction of the near-peer and far-peer GPA variables. A. Histograms of the proportion of peers within each school that constituted near (blue) and far (red) peer variables. B. Histograms of the absoolute number of peers within each school that constituted near (blue) and far (red) peer variables. C. Example similarity matrix depicting the number of shared core courses between any two students. Each colored square is a pair of students. The diagonal represents the number of core courses each student is taking. Lighter colors represent more shared core courses. . . . . 7
S2 Within- and between-school variation in conscientiousness and self-regulation standards. Each black dot is an individual student, arranged in vertical stacks, each of which represents a school. The red dots are the school-wide means for each variable. . . . . 7
S3 In the Academic Diligence Task (1), students choose between "Do math" or "Play game or watch movie." If they click "Do math," they solve single-digit subtraction problems. If they instead click "Play game or watch movie," a pull-down menu is displayed that contains various video clips or the option to play the video game Tetris. At any point the students can toggle between math or entertainment, but the program restricts engagement to one activity at a time. Figure reproduced with permission from Galla et al. (1). . . . . 16


## List of Tables


S1 Demographic variables included as controls in OLS models in Study 1. . . . . 3
S2 Descriptive statistics, bivariate correlations, and partial correlations for Study 1 . . . . . 3
S3 OLS regression models predicting self-reported grit from self and peer academic performance in Study 1 . . . . 4
S4 Subgroup analyses by range of individual GPA in Study 1 . . . . . 4
S5 Subgroup analyses by individual performance quintiles in Study 1 . . . . . 5
S6 Subgroup analyses by gender, school type, and mother's education in Study 1 . . . . . 5
S7 Subgroup analyses by school size tertiles in Study 1 . . . . . 6
S8 Means, standard deviations, and correlation coefficients among the study variables in Study 2 . . . . . 8
S9 Differences across schools and intraclass correlation coefficients in Study 2 . . . . . 8
S10 OLS regression models predicting self-reported conscientiousness and self-regulation standards, respectively, in Study 2 . . . . . 9
S11 Subgroup analyses by gender in Study 2 . . . . . 10
S12 Subgroup analyses by grade in Study 2 . . . . . 11
S13 Subgroup analyses by eligibility for free or reduced priced meals status in Study 2 . . . . . 12
S14 Subgroup analyses by race/ethnicity in Study 2 . . . . . 13
S15 Subgroup analyses by English language learner status in Study 2 . . . . . 14
S16 Subgroup analyses by GPA tertiles in Study 2 . . . . . 15
S17 Means, standard deviations, and correlation coefficients in Study 3. . . . . 16
S18 Multilevel logistic regression models predicting college graduation using the full sample in Study 3 . . . . . 17
S19 Models Predicting College Graduation from Self-Reported Grit and Self-Control, and the Academic Diligence Task (untransformed) . . . . . 18
S20 Multilevel logistic regression models predicting college graduation using listwise deletion in Study 3 . . . . . 19
S21 OLS regression models predicting self-reported grit and self-control, and the Academic Diligence Task from peer and own performance in Study 3 (conceptual replication of Studies 1 and 2). . . . . 20

## Supporting Information Text

## 1. Study 1

**Table S1. Demographic variables included as controls in OLS models in Study 1.**

| **Question** |
|---|
| Gender |
| How old are you? |
| Marital status |
| Does your mother speak an indigenous language or dialect? |
| Does your father speak an indigenous language or dialect? |
| Do you live with your mother? |
| Do you live with your father? |
| When you have an academic doubt, do you have someone to help you? |
| What is the level of education reached by your mother (even if she passed away)? |
| What is the level of education reached by your father (even if he passed away)? |
| How many books are there in your house? (Don't include magazines, newspapers, or textbooks.) |
| In the last 2 years, how many times have you gone on vacation within the Mexican Republic? |
| How many states of the Mexican Republic have you visited as a tourist? |
| At home, do you have a conventional telephone (landline)? |
| At home, do you have a cell phone? |
| At home, do you have a washing machine? |
| At home, do you have a refrigerator? |
| At home, do you have a gas stove? |
| At home, do you have a microwave oven? |
| At home, do you have cable or satellite TV? |
| At home, do you have Internet? |
| How many DVD players are there in your house? |
| How many computers are there in your house? |
| How many televisions are there in your house? |
| How many cars are there in your house? |

**Table S2. Descriptive statistics, bivariate correlations, and partial correlations for Study 1**

| | 1 | 2 | 3 | 4 | 5 | 6 | 7 | 8 | 9 | 10 | 11 | 12 |
|---|---|---|---|---|---|---|---|---|---|---|---|---|
| *Own* | | | | | | | | | | | | |
| 1. Grit | | 0.28*** | 0.25*** | 0.25*** | 0.13*** | 0.15*** | 0.12*** | −0.02*** | −0.01*** | −0.03*** | 0.01** | 0.01*** |
| 2. Overall GPA | 0.26*** | | 0.61*** | 0.57*** | 0.36*** | 0.36*** | −0.01*** | 0.03*** | 0.02*** | 0.02*** | 0.00* | 0.00 |
| 3. Math GPA | 0.24*** | 0.65*** | | 0.43*** | 0.42*** | 0.29*** | 0.00* | 0.02*** | 0.04*** | 0.01*** | 0.01* | 0.00 |
| 4. Verbal GPA | 0.25*** | 0.61*** | 0.46*** | | 0.20*** | 0.30*** | −0.02*** | 0.02*** | 0.01*** | 0.04*** | 0.01*** | 0.01*** |
| 5. Math test score | 0.14*** | 0.29*** | 0.36*** | 0.19*** | | 0.53*** | 0.01** | 0.01** | 0.01*** | 0.01*** | 0.13*** | 0.07*** |
| 6. Reading test score | 0.16*** | 0.31*** | 0.27*** | 0.29*** | 0.63*** | | 0.01*** | 0.00 | 0.00 | 0.01*** | 0.07*** | 0.11*** |
| *Peers'* | | | | | | | | | | | | |
| 7. Grit | 0.21*** | 0.05*** | 0.05*** | 0.08*** | 0.12*** | 0.14*** | | 0.01*** | 0.04*** | −0.09*** | 0.08*** | 0.12*** |
| 8. Overall GPA | 0.03*** | 0.45*** | 0.31*** | 0.30*** | 0.04*** | 0.07*** | 0.13*** | | 0.58*** | 0.59*** | 0.18*** | 0.15*** |
| 9. Math GPA | 0.03*** | 0.37*** | 0.36*** | 0.26*** | 0.08*** | 0.09*** | 0.14*** | 0.83*** | | 0.37*** | 0.20*** | 0.12*** |
| 10. Verbal GPA | 0.05*** | 0.35*** | 0.24*** | 0.39*** | 0.10*** | 0.14*** | 0.22*** | 0.77*** | 0.65*** | | 0.16*** | 0.22*** |
| 11. Math test score | 0.05*** | 0.04*** | 0.05*** | 0.07*** | 0.55*** | 0.46*** | 0.22*** | 0.08*** | 0.15*** | 0.18*** | | 0.59*** |
| 12. Reading test score | 0.06*** | 0.06*** | 0.07*** | 0.11*** | 0.49*** | 0.51*** | 0.28*** | 0.14*** | 0.19*** | 0.27*** | 0.89*** | |
| *M* | 3.72 | 8.38 | 8.05 | 8.70 | 0.10 | 0.10 | 3.72 | 8.38 | 8.05 | 8.70 | 0.10 | 0.10 |
| *SD* | 0.56 | 0.73 | 1.11 | 0.85 | 1.00 | 1.01 | 0.13 | 0.33 | 0.42 | 0.34 | 0.56 | 0.52 |
| *N* | 206,589 | 210,423 | 201,445 | 190,658 | 211,255 | 211,255 | 206,589 | 210,423 | 201,445 | 190,658 | 211,255 | 211,255 |

*Notes.* Bivariate correlations under diagonal. Partial correlations controlling for school dummy variables shown above the diagonal. Missing data was handled with pairwise deletion.

* p < .05, ** p < .01, *** p < .001.

**Table S3. OLS regression models predicting self-reported grit from self and peer academic performance in Study 1**

| | Overall GPA | Math GPA | Language GPA | Math Test Score | Reading Test Score |
|---|---|---|---|---|---|
| Own performance | 0.427*** | 0.233*** | 0.308*** | 0.163*** | 0.156*** |
| | (0.005) | (0.004) | (0.004) | (0.003) | (0.004) |
| Peer performance | −0.245*** | −0.133*** | −0.196*** | −0.096*** | −0.062** |
| | (0.051) | (0.028) | (0.039) | (0.022) | (0.022) |
| $R^2$ | 0.124 | 0.110 | 0.109 | 0.071 | 0.071 |
| *N* | 205,901 | 197,139 | 186,551 | 206,589 | 206,589 |

*Notes.* All OLS regression models include high school fixed effects, year dummies, and controls for student characteristics. Robust standard errors clustered by high school. The number of observations differs across regressions because of missing values. Own and Peer performance refer to Overall GPA, Math GPA, Language GPA, Math Test Score, and Reading Test Score, respectively, depending on the model. For example, the first column summarises an OLS regression model predicting self-reported grit from own overall GPA ($\beta$ = 0.427) and peer overall GPA ($\beta$ = -0.245).
* p < .05, ** p < .01, *** p < .001.

**Table S4. Subgroup analyses by range of individual GPA in Study 1**

| Individual GPA Range | Peer-Average GPA | Peer-Average Language GPA | Peer-Average Math GPA |
|---|---|---|---|
| <6 | | 0.599 | 0.211 |
| | | (1.250) | (0.176) |
| 6.0 - 6.4 | 0.744 | −0.511 | −0.101 |
| | (0.855) | (0.374) | (0.067) |
| 6.5 - 6.9 | 0.193 | −0.016 | −0.093 |
| | (0.303) | (0.288) | (0.093) |
| 7.0 - 7.4 | −0.132 | −0.250* | −0.162** |
| | (0.132) | (0.124) | (0.061) |
| 7.5 - 7.9 | −0.172 | −0.158 | −0.136** |
| | (0.088) | (0.109) | (0.065) |
| 8.0 - 8.4 | −0.224** | −0.140** | −0.141** |
| | (0.074) | (0.058) | (0.051) |
| 8.5 - 8.9 | −0.312*** | −0.229** | −0.125** |
| | (0.088) | (0.072) | (0.062) |
| 9.0 - 9.4 | −0.459*** | −0.280*** | −0.324*** |
| | (0.102) | (0.075) | (0.063) |
| 9.5 - 9.9 | −0.126 | −0.241 | −0.112 |
| | (0.153) | (0.130) | (0.114) |
| 10 | −0.937 | −0.125 | 0.095 |
| | (0.948) | (0.118) | (0.113) |

*Notes.* Models additionally include parameters for individuals own GPA, controls for student characteristics, and fixed effects for school and class year, not shown. Robust standard errors clustered by high school.Each cell summarises a model, with the coefficient being shown is the effect of peer performance (operationalized as the column header), in the particular subset of the data specified by the row (e.g., the $\beta$ = 0.599 in the second column and first row is the effect of peer-average language GPA on self-reported grit in the subset of students who reported a Language GPA of 6 or lower.
* p < .05, ** p < .01, *** p < .001.

**Table S5. Subgroup analyses by individual performance quintiles in Study 1**

| Individual Test Score Quintile | Math Test Score | Reading Test Score |
|---|---|---|
| Bottom 20% | −0.062 | −0.037 |
| | (0.043) | (0.039) |
| Quintile 2 | −0.031 | −0.040 |
| | (0.041) | (0.042) |
| Quintile 3 | −0.106** | −0.064 |
| | (0.040) | (0.039) |
| Quintile 4 | −0.130** | −0.033 |
| | (0.041) | (0.040) |
| Top 20% | −0.102* | −0.100 |
| | (0.050) | (0.054) |

*Notes.* Models additionally include parameters for individuals own test scores, controls for student characteristics, and fixed effects for school and class year, not shown. Robust standard errors clustered by high school. Each cell summarises a model, with the coefficient being shown is the effect of peer performance (operationalized as the column header), in the particular subset of the data specified by the row (e.g., the $\beta$ = 0.-0.062 in the first column and first row is the effect of peer-average math test score on self-reported grit in the subset of students who reported a scored in the bottom 20% of the math test score distribution.

† p < .10, * p < .05, ** p < .01, *** p < .001.

**Table S6. Subgroup analyses by gender, school type, and mother's education in Study 1**

| | Overall GPA | | Math GPA | | Language GPA | | Math Test Score | | Reading Test Score | |
|---|---|---|---|---|---|---|---|---|---|---|
| | (1) | (2) | (1) | (2) | (1) | (2) | (1) | (2) | (1) | (2) |
| **Student gender: boys (1) vs. girls (2)** | | | | | | | | | | |
| Own performance | 0.390*** | 0.466*** | 0.222*** | 0.246*** | 0.277*** | 0.342*** | 0.141*** | 0.185*** | 0.135*** | 0.181*** |
| | (0.006) | (0.006) | (0.004) | (0.005) | (0.005) | (0.006) | (0.004) | (0.005) | (0.004) | (0.005) |
| Peer performance | −0.207** | −0.275*** | −0.146*** | −0.124*** | −0.226*** | −0.175*** | −0.063** | −0.135*** | −0.055 | −0.076** |
| | (0.066) | (0.061) | (0.039) | (0.034) | (0.051) | (0.051) | (0.029) | (0.029) | (0.029) | (0.029) |
| $R^2$ | 0.112 | 0.139 | 0.102 | 0.121 | 0.099 | 0.122 | 0.064 | 0.080 | 0.064 | 0.080 |
| *n* | 95,447 | 110,400 | 92,472 | 104,616 | 87,218 | 99,282 | 95,780 | 110,755 | 95,780 | 110,755 |
| **School type: public (1) vs. private (2)** | | | | | | | | | | |
| Own performance | 0.421*** | 0.454*** | 0.231*** | 0.241*** | 0.302*** | 0.338*** | 0.161*** | 0.171*** | 0.156*** | 0.157*** |
| | (0.005) | (0.011) | (0.004) | (0.007) | (0.004) | (0.009) | (0.004) | (0.008) | (0.004) | (0.006) |
| Peer performance | −0.255*** | −0.242** | −0.132*** | −0.096 | −0.182*** | −0.206** | −0.101*** | −0.078 | −0.056* | −0.130** |
| | (0.059) | (0.089) | (0.031) | (0.059) | (0.043) | (0.076) | (0.024) | (0.046) | (0.024) | (0.044) |
| $R^2$ | 0.123 | 0.135 | 0.111 | 0.113 | 0.108 | 0.115 | 0.072 | 0.073 | 0.072 | 0.073 |
| *n* | 170,188 | 35,713 | 162,280 | 34,859 | 153,070 | 33,481 | 170,769 | 35,820 | 170,769 | 35,820 |
| **Mothers education: less than high school (1) vs. more than high school (2)** | | | | | | | | | | |
| Own performance | 0.424*** | 0.428*** | 0.236*** | 0.229*** | 0.304*** | 0.311*** | 0.175*** | 0.149*** | 0.174*** | 0.138*** |
| | (0.006) | (0.006) | (0.004) | (0.004) | (0.005) | (0.005) | (0.004) | (0.005) | (0.005) | (0.005) |
| Peer performance | −0.207*** | −0.251*** | −0.072* | −0.186*** | −0.162*** | −0.214*** | −0.109*** | −0.074*** | −0.062* | −0.056* |
| | (0.062) | (0.071) | (0.035) | (0.037) | (0.049) | (0.052) | (0.025) | (0.031) | (0.028) | (0.029) |
| $R^2$ | 0.130 | 0.124 | 0.117 | 0.109 | 0.116 | 0.108 | 0.079 | 0.069 | 0.080 | 0.069 |
| *n* | 104,058 | 100,531 | 98,866 | 97,066 | 93,280 | 92,127 | 104,452 | 100,803 | 104,452 | 100,803 |

*Notes.* All regressions include high school fixed effects, year dummies, and controls for student characteristics not used in the subgroup analyses. Robust standard errors clustered by high school. Own and Peer performance refer to Overall GPA, Math GPA, Language GPA, Math Test Score, and Reading Test Score, respectively, depending on the model. For example, the first column summarises an OLS regression model predicting self-reported grit from own overall GPA ($\beta$ = 0.390) and peer overall GPA ($\beta$ = -0.207) in the subset of boys. We conducted tests of equality of the regression coefficients (2) for each pair of models (e.g., effect of peer overall GPA for boys and girls), and found that all pairs of coefficients were statistically indistinguishable from each other (all *p*s > .05, after correcting for multiple comparisons with the Benjamini-Hochberg False Discovery Rate correction (3)

* p < .05, ** p < .01, *** p < .001.

**Table S7. Subgroup analyses by school size tertiles in Study 1**

| | Smallest third | Middle third | Largest third |
|---|---|---|---|
| **Overall GPA** | | | |
| Own performance | 0.436*** | 0.438*** | 0.421*** |
| | (0.012) | (0.008) | (0.006) |
| Peer performance | −0.152 | −0.118 | −0.357*** |
| | (0.085) | (0.087) | (0.081) |
| $R^2$ | 0.150 | 0.130 | 0.115 |
| *n* | 19,957 | 57,676 | 128,268 |
| **Math GPA** | | | |
| Own performance | 0.258*** | 0.251*** | 0.222*** |
| | (0.008) | (0.005) | (0.005) |
| Peer performance | −0.060 | −0.126** | −0.167*** |
| | (0.055) | (0.047) | (0.044) |
| $R^2$ | 0.138 | 0.116 | 0.101 |
| *n* | 19,023 | 54,254 | 123,862 |
| **Language GPA** | | | |
| Own performance | 0.328*** | 0.325*** | 0.298*** |
| | (0.011) | (0.007) | (0.005) |
| Peer performance | −0.121 | −0.138 | −0.270*** |
| | (0.071) | (0.074) | (0.058) |
| $R^2$ | 0.131 | 0.115 | 0.099 |
| *n* | 18,250 | 51,242 | 117,059 |
| **Math Test Scores** | | | |
| Own performance | 0.202*** | 0.176*** | 0.151*** |
| | (0.010) | (0.006) | (0.004) |
| Peer performance | −0.158*** | −0.090* | −0.086* |
| | (0.040) | (0.035) | (0.037) |
| $R^2$ | 0.094 | 0.073 | 0.063 |
| *n* | 20,057 | 57,950 | 128,582 |
| **Reading Test Scores** | | | |
| Own performance | 0.191*** | 0.175*** | 0.143*** |
| | (0.010) | (0.006) | (0.005) |
| Peer performance | −0.156** | −0.033 | −0.054 |
| | (0.046) | (0.035) | (0.032) |
| $R^2$ | 0.0795 | 0.075 | 0.063 |
| *n* | 20,057 | 57,950 | 128,582 |

*Notes.* Models additionally include parameters for individuals own GPA, controls for student characteristics, and fixed effects for school and class year, not shown. Robust standard errors clustered by high school. Each column summarises 5 models, one for each operationalization of own and peer performance (shown in bold headers) predicting self-reported grit. Each column represents a subset of the data based on school size (e.g., the $\beta$ = 0.328 in the first column and first row is the effect of a student's own overall GPA on self-reported grit in the subset of students who attend the third of the smallest schools in our dataset.
* p < 0.05, ** p < 0.01, *** p < 0.001.

## 2. Study 2

**A. Note on items assessing self-regulation standards.** We created two standards items for this investigation based on our prior studies of self-regulation in adolescence. Specifically, following the item "I forgot something I needed for class" from the Domain-Specific Impulsivity Scale (4) inspired this question: "If a student in your grade says they 'sometimes' forget something they need for class, how often would you guess they mean?" Seven response options ranged from *once a month* to *three times or more per day.* Likewise, hours of homework was a focal measure in "Self-Discipline Outdoes IQ in Predicting Academic Performance of Adolescents" (5) and inspired this question: "If a student in your grade says they did 'a lot of homework' on a weeknight, how long would you guess they mean?" Eight response options ranged from 15 minutes to 3 or more hours.

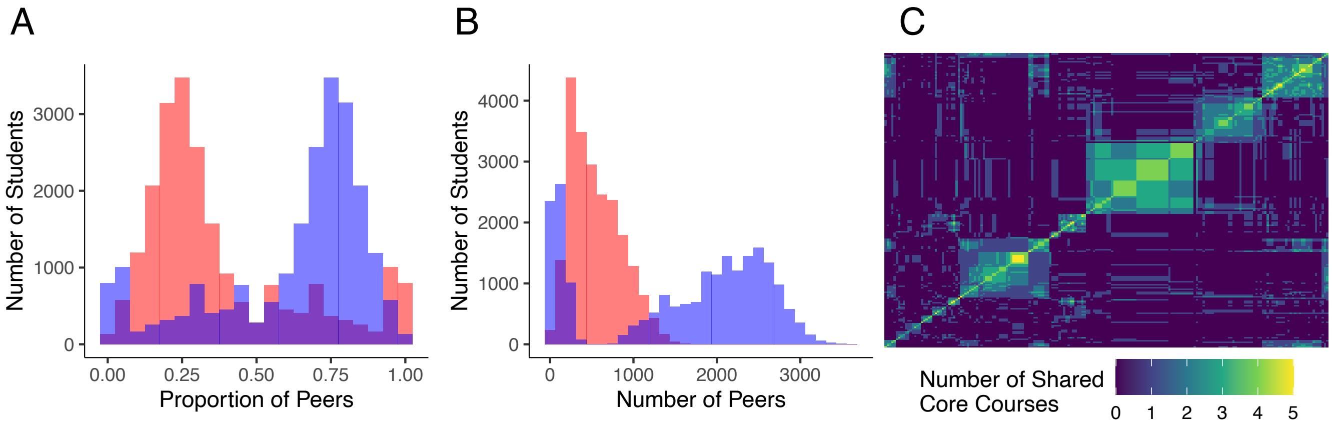


**Fig. S1.** Details about the construction of the near-peer and far-peer GPA variables. A. Histograms of the proportion of peers within each school that constituted near (blue) and far (red) peer variables. B. Histograms of the absoulute number of peers within each school that constituted near (blue) and far (red) peer variables. C. Example similarity matrix depicting the number of shared core courses between any two students. Each colored square is a pair of students. The diagonal represents the number of core courses each student is taking. Lighter colors represent more shared core courses.

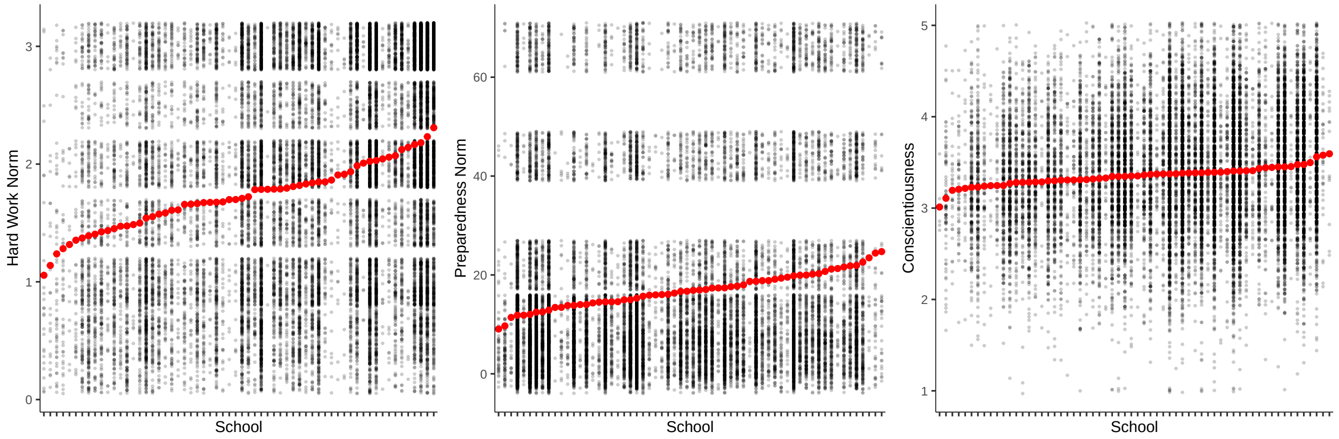


**Fig. S2.** Within- and between-school variation in conscientiousness and self-regulation standards. Each black dot is an individual student, arranged in vertical stacks, each of which represents a school. The red dots are the school-wide means for each variable.

**Table S8. Means, standard deviations, and correlation coefficients among the study variables in Study 2**

| | 1 | 2 | 3 | 4 | 5 | 6 | 7 |
|---|---|---|---|---|---|---|---|
| **Own** | | | | | | | |
| 1. Preparedness norm | - | 0.15*** | 0.06*** | 0.11*** | - | 0.15*** | −0.10*** |
| 2. Hard work norm | 0.18*** | - | 0.09*** | 0.19*** | - | 0.25*** | −0.14*** |
| 3. Conscientiousness | 0.06*** | 0.10*** | - | 0.25*** | - | 0.05*** | −0.02*** |
| 4. Core GPA | 0.13*** | 0.19*** | 0.24*** | - | - | 0.42*** | −0.28*** |
| **Peer** | | | | | | | |
| 5. Core GPA (in same school) | 0.09*** | 0.10*** | −0.02** | 0.22*** | - | - | - |
| 6. Core GPA (peers in shared core courses) | 0.17*** | 0.25*** | 0.03*** | 0.46*** | 0.62*** | - | −0.54*** |
| 7. Core GPA (peers not in shared core courses) | −0.02** | −0.07*** | −0.05*** | −0.08*** | 0.55*** | 0.02*** | - |
| **Student demographics** | | | | | | | |
| Female | 0.03*** | 0.12*** | 0.09*** | 0.15*** | −0.02** | 0.04*** | −0.04*** |
| Age | 0.03*** | 0.12*** | 0.09*** | −0.04*** | −0.10*** | 0.00 | −0.16*** |
| English language learner | −0.04*** | −0.05*** | 0.03*** | −0.12*** | −0.04*** | −0.26*** | 0.15*** |
| Special education student | 0.02* | 0.03*** | −0.03*** | 0.03*** | 0.04*** | 0.05*** | 0.04*** |
| Eligible for free or reduced-price meals | −0.12*** | −0.16*** | −0.01 | −0.24*** | −0.19*** | −0.30*** | −0.02* |
| **Race/ethnicity** | | | | | | | |
| Hispanic | −0.05*** | −0.06*** | −0.02** | −0.11*** | 0.09*** | −0.10*** | 0.17*** |
| Black, non-Hispanic | −0.09*** | −0.10*** | 0.03*** | −0.13*** | −0.35*** | −0.25*** | −0.21*** |
| White, non-Hispanic | 0.12*** | 0.12*** | −0.01 | 0.16*** | 0.20*** | 0.27*** | 0.03*** |
| Other | 0.04*** | 0.07*** | 0.01 | 0.13*** | 0.05*** | 0.13*** | −0.03*** |
| **Grade level** | | | | | | | |
| 8 | −0.06*** | −0.17*** | −0.07*** | 0.04*** | 0.07*** | 0.07*** | 0.18*** |
| 9 | 0.03*** | 0.02* | −0.02*** | −0.01 | 0.05*** | −0.10*** | 0.02* |
| 10 | −0.02** | 0.01 | −0.01 | −0.06*** | −0.10*** | −0.11*** | −0.09*** |
| 11 | 0.03*** | 0.10*** | 0.05*** | 0.00 | 0.00 | 0.05*** | −0.06*** |
| 12 | 0.03*** | 0.07*** | 0.06*** | 0.04*** | −0.04*** | 0.09*** | −0.08*** |
| **Home language** | | | | | | | |
| English | 0.06*** | 0.05*** | 0.00 | 0.06*** | 0.04*** | 0.15*** | −0.07*** |
| Spanish | −0.06*** | −0.06*** | 0.00 | −0.09*** | 0.03*** | −0.14*** | 0.13*** |
| Other | 0.00 | 0.00 | 0.01 | 0.03*** | −0.10*** | −0.04*** | −0.08*** |
| Took survey in winter term (versus fall term) | −0.03*** | −0.08*** | −0.07*** | −0.02** | −0.16*** | −0.08*** | −0.08*** |
| *M* | 50.13 | 1.90 | 3.39 | 80.97 | 80.10 | 80.01 | 80.60 |
| *SD* | 18.82 | 0.93 | 0.62 | 10.36 | 2.76 | 4.18 | 4.20 |
| *N* | 21,818 | 21,818 | 21,818 | 21,818 | 21,818 | 21,818 | 21,818 |

*Notes.* Bivariate correlations appear below the diagonal. Partial Correlations controlling for school dummy variables appear above the diagonal. We do not show partial correlations between peer GPA and other variables because the peer terms are collinear with the school dummy indicators.
* p < .05, ** p < .01, *** p < .001.

**Table S9. Differences across schools and intraclass correlation coefficients in Study 2**

| | | Not controlling for student demographics | | Controlling for student demographics | | |
|---|---|---|---|---|---|---|
| | Kurtosis | $R^2$ | *F* | $R^2$ | *F* | ICC |
| Conscientiousness | 1.083 | 0.015 | 5.562*** | 0.030 | 8.905*** | 0.013 |
| Hard work norm | −0.525 | 0.079 | 30.733*** | 0.118 | 38.361*** | 0.077 |
| Preparedness norm | −0.598 | 0.030 | 10.904*** | 0.043 | 12.724*** | 0.026 |
| **Big Five Conscientiousness items** | | | | | | |
| Reliable | −0.688 | 0.010 | 3.475*** | 0.038 | 11.421*** | 0.008 |
| Irresponsible | −0.587 | 0.012 | 4.155*** | 0.029 | 8.661*** | 0.010 |
| Dependable | −0.308 | 0.009 | 3.294*** | 0.021 | 6.272*** | 0.007 |
| Careless | −0.177 | 0.006 | 2.127*** | 0.014 | 4.201*** | 0.003 |
| Difficulty starting | 0.129 | 0.016 | 5.824*** | 0.023 | 6.633*** | 0.012 |
| Lazy | 0.729 | 0.011 | 4.065*** | 0.021 | 6.190*** | 0.010 |
| Tidy | 2.007 | 0.012 | 4.380*** | 0.029 | 8.624*** | 0.009 |
| Systematic | 2.033 | 0.008 | 3.002*** | 0.021 | 6.225*** | 0.006 |
| Messy | 2.302 | 0.018 | 6.473*** | 0.023 | 6.793*** | 0.014 |
| Persistent | 2.676 | 0.009 | 3.147*** | 0.017 | 4.829*** | 0.007 |
| Disorganized | 2.725 | 0.008 | 2.910*** | 0.016 | 4.693*** | 0.005 |
| Efficient | 4.526 | 0.007 | 2.612*** | 0.014 | 4.167*** | 0.005 |

*Notes.* Bootstrapped confidence intervals with *B* = 1000 replications show that there is significantly more between-school variation for both standards (95% CIs [0.071 – 0.096] and [0.025 – 0.041], for hard work and preparedness norms respectively) for self-regulation compared to conscientiousness (95% CI [0.013 – 0.023]).
* p < .05, ** p < .01, *** p < .001.

**Table S10. OLS regression models predicting self-reported conscientiousness and self-regulation standards, respectively, in Study 2**

| | Conscientiousness | | | | | Hard work norm | | | | | Preparedness norm | | | | |
|---|---|---|---|---|---|---|---|---|---|---|---|---|---|---|---|
| | (1) | (2) | (3) | (4) | (5) | (1) | (2) | (3) | (4) | (5) | (1) | (2) | (3) | (4) | (5) |
| Own GPA | 0.258*** | 0.288*** | 0.258*** | 0.288*** | 0.292*** | 0.183*** | 0.097*** | 0.183*** | 0.097*** | 0.071*** | 0.113*** | 0.058*** | 0.113*** | 0.058*** | 0.045*** |
| | (0.007) | (0.007) | (0.012) | (0.012) | (0.013) | (0.007) | (0.007) | (0.011) | (0.008) | (0.007) | (0.007) | (0.008) | (0.011) | (0.009) | (0.009) |
| School-peer GPA | 0.082** | | 0.082 | | | −0.015 | | −0.015 | | | −0.015 | | −0.015 | | |
| | (0.030) | | (0.041) | | | (0.030) | | (0.046) | | | (0.031) | | (0.033) | | |
| Near-peer GPA | | −0.065*** | | −0.065*** | −0.059*** | | 0.248*** | | 0.248*** | 0.230*** | | 0.150*** | | 0.150*** | 0.143*** |
| | | (0.011) | | (0.015) | (0.016) | | (0.010) | | (0.025) | (0.024) | | (0.011) | | (0.013) | (0.013) |
| Far-peer GPA | | 0.038** | | 0.038* | 0.012 | | −0.011 | | −0.011 | −0.025 | | −0.023 | | −0.023* | −0.021 |
| | | (0.012) | | (0.016) | (0.014) | | (0.011) | | (0.020) | (0.020) | | (0.012) | | (0.011) | (0.012) |
| **Race/ethnicity** | | | | | | | | | | | | | | | |
| White, non-Hispanic (reference) | | | | | | | | | | | | | | | |
| Black, non-Hispanic | | | | | 0.034*** | | | | | −0.030** | | | | | −0.053*** |
| | | | | | (0.009) | | | | | (0.009) | | | | | (0.007) |
| Hispanic | | | | | 0.018 | | | | | −0.016 | | | | | −0.035*** |
| | | | | | (0.011) | | | | | (0.010) | | | | | (0.009) |
| Other race | | | | | 0.001 | | | | | 0.007 | | | | | −0.011 |
| | | | | | (0.008) | | | | | (0.007) | | | | | (0.007) |
| **Grade level** | | | | | | | | | | | | | | | |
| Grade 8 (reference) | | | | | | | | | | | | | | | |
| Grade 9 | | | | | −0.030 | | | | | −0.029 | | | | | 0.059 |
| | | | | | (0.077) | | | | | (0.024) | | | | | (0.073) |
| Grade 10 | | | | | −0.041 | | | | | 0.024 | | | | | 0.041 |
| | | | | | (0.073) | | | | | (0.023) | | | | | (0.067) |
| Grade 11 | | | | | −0.034 | | | | | 0.080** | | | | | 0.058 |
| | | | | | (0.084) | | | | | (0.028) | | | | | (0.077) |
| Grade 12 | | | | | −0.037 | | | | | 0.079** | | | | | 0.045 |
| | | | | | (0.070) | | | | | (0.026) | | | | | (0.063) |
| **Student demographics** | | | | | | | | | | | | | | | |
| Female | | | | | 0.045*** | | | | | 0.096*** | | | | | 0.019** |
| | | | | | (0.007) | | | | | (0.007) | | | | | (0.007) |
| Eligible for free or reduced priced meals | | | | | 0.024** | | | | | −0.043*** | | | | | −0.032*** |
| | | | | | (0.007) | | | | | (0.010) | | | | | (0.006) |
| English language learner | | | | | 0.052*** | | | | | 0.036*** | | | | | 0.020* |
| | | | | | (0.008) | | | | | (0.007) | | | | | (0.009) |
| Special education student | | | | | −0.026*** | | | | | 0.008 | | | | | −0.006 |
| | | | | | (0.007) | | | | | (0.007) | | | | | (0.007) |
| Took survey in the winter term | | | | | −0.044*** | | | | | −0.017 | | | | | 0.008 |
| | | | | | (0.009) | | | | | (0.014) | | | | | (0.009) |
| Age | | | | | 0.105*** | | | | | −0.115*** | | | | | −0.016 |
| | | | | | (0.015) | | | | | (0.017) | | | | | (0.021) |
| **Home language** | | | | | | | | | | | | | | | |
| English (reference) | | | | | | | | | | | | | | | |
| Spanish | | | | | 0.008 | | | | | 0.007 | | | | | −0.015 |
| | | | | | (0.012) | | | | | (0.010) | | | | | (0.010) |
| Other | | | | | −0.027* | | | | | −0.002 | | | | | −0.0001 |
| | | | | | (0.010) | | | | | (0.006) | | | | | (0.008) |
| *N* | 21,818 | 21,818 | 21,818 | 21,818 | 21,818 | 21,818 | 21,818 | 21,818 | 21,818 | 21,818 | 21,818 | 21,818 | 21,818 | 21,818 | 21,818 |
| $R^2$ | 0.079 | 0.082 | 0.079 | 0.082 | 0.095 | 0.111 | 0.143 | 0.111 | 0.143 | 0.159 | 0.042 | 0.055 | 0.042 | 0.055 | 0.059 |

*Notes.* For each variable we report 5 models. (1) School-wide peers and no student characteristics, (2) Near- and far-peers and no student characteristics, (3) School-wide peers, no student characteristics, and school fixed-effects, (4) Near- and far-peers, no student characteristics, and school fixed-effects, (5) Near- and far-peers, student characteristics, and school fixed-effects. Robust standard errors clustered by school.

*Notes.* * p < .05, ** p < .01, *** p < .001.

**Table S11. Subgroup analyses by gender in Study 2**

| | Male | | | Female | | |
|---|---|---|---|---|---|---|
| | C | HWN | PN | C | HWN | PN |
| Own GPA | 0.288*** | 0.078*** | 0.057*** | 0.293*** | 0.066*** | 0.033** |
| | (0.013) | (0.011) | (0.013) | (0.015) | (0.010) | (0.010) |
| Near-peer GPA | −0.089*** | 0.187*** | 0.103*** | −0.029 | 0.280*** | 0.187*** |
| | (0.019) | (0.027) | (0.019) | (0.018) | (0.026) | (0.015) |
| Far-peer GPA | 0.011 | −0.043* | −0.030 | 0.009 | −0.009 | −0.010 |
| | (0.019) | (0.018) | (0.018) | (0.018) | (0.027) | (0.018) |
| **Race/ethnicity** | | | | | | |
| White, non-Hispanic (reference) | | | | | | |
| Black, non-Hispanic | 0.041*** | −0.038** | −0.052*** | 0.028 | −0.021 | −0.053*** |
| | (0.010) | (0.013) | (0.014) | (0.016) | (0.013) | (0.012) |
| Hispanic | 0.032 | −0.015 | −0.046** | 0.003 | −0.017 | −0.026* |
| | (0.019) | (0.012) | (0.015) | (0.017) | (0.014) | (0.011) |
| Other race | 0.016 | 0.005 | −0.021* | −0.015 | 0.007 | −0.002 |
| | (0.009) | (0.011) | (0.010) | (0.011) | (0.008) | (0.011) |
| **Grade level** | | | | | | |
| Grade 8 (reference) | | | | | | |
| Grade 9 | −0.087 | −0.052 | 0.126 | 0.030 | −0.018 | 0.009 |
| | (0.118) | (0.080) | (0.131) | (0.073) | (0.039) | (0.060) |
| Grade 10 | −0.089 | −0.009 | 0.108 | 0.007 | 0.043 | −0.009 |
| | (0.111) | (0.076) | (0.121) | (0.074) | (0.034) | (0.059) |
| Grade 11 | −0.087 | 0.064 | 0.117 | 0.020 | 0.082 | 0.021 |
| | (0.126) | (0.088) | (0.137) | (0.087) | (0.041) | (0.071) |
| Grade 12 | −0.067 | 0.078 | 0.071 | −0.003 | 0.070 | 0.037 |
| | (0.105) | (0.072) | (0.113) | (0.074) | (0.041) | (0.062) |
| **Student demographics** | | | | | | |
| Eligible for free or reduced priced meals | 0.035*** | −0.049** | −0.048*** | 0.014 | −0.036*** | −0.016 |
| | (0.010) | (0.015) | (0.012) | (0.011) | (0.009) | (0.010) |
| English language learner | 0.070*** | 0.040*** | 0.037*** | 0.037*** | 0.035** | 0.004 |
| | (0.010) | (0.011) | (0.010) | (0.010) | (0.011) | (0.012) |
| Special education student | −0.034** | 0.007 | −0.006 | −0.015 | 0.010 | −0.005 |
| | (0.010) | (0.008) | (0.009) | (0.010) | (0.012) | (0.009) |
| Took survey in the winter term | −0.036* | −0.029 | 0.005 | −0.055*** | −0.003 | 0.014 |
| | (0.014) | (0.015) | (0.013) | (0.012) | (0.017) | (0.012) |
| Age | 0.090** | −0.151*** | 0.029 | 0.120*** | −0.081*** | −0.063 |
| | (0.028) | (0.025) | (0.021) | (0.026) | (0.022) | (0.034) |
| **Home language** | | | | | | |
| English (reference) | | | | | | |
| Spanish | 0.004 | −0.009 | −0.010 | 0.014 | 0.024 | −0.017 |
| | (0.015) | (0.013) | (0.015) | (0.015) | (0.016) | (0.012) |
| Other | −0.025 | 0.005 | −0.010 | −0.031* | −0.009 | 0.011 |
| | (0.013) | (0.008) | (0.010) | (0.013) | (0.009) | (0.008) |
| *n* | 10,868 | 10,868 | 10,868 | 10,950 | 10,950 | 10,950 |
| $R^2$ | 0.088 | 0.133 | 0.053 | 0.099 | 0.169 | 0.074 |

*Notes* Estimates calculated by running separate OLS regressions for each subgroup. Robust standard errors clustered by school. C = Conscientiousness, HWN = Hard work norm, PN = Preparedness norm.
* p < .05, ** p < .01, *** p < .001.

**Table S12. Subgroup analyses by grade in Study 2**

| | Grade 8 | | | Grade 9 | | | Grade 10 | | | Grade 11 | | | Grade 12 | | |
|---|---|---|---|---|---|---|---|---|---|---|---|---|---|---|---|
| | C | HWN | PN | C | HWN | PN | C | HWN | PN | C | HWN | PN | C | HWN | PN |
| Own GPA | 0.334*** | 0.091*** | 0.051** | 0.328*** | 0.076*** | 0.065* | 0.300*** | 0.089*** | 0.042* | 0.260*** | 0.044** | 0.036 | 0.231*** | 0.043 | 0.029* |
| | (0.016) | (0.013) | (0.015) | (0.014) | (0.014) | (0.025) | (0.016) | (0.022) | (0.018) | (0.021) | (0.014) | (0.018) | (0.021) | (0.027) | (0.013) |
| Near-peer GPA | −0.080* | 0.074 | 0.079** | −0.060* | 0.203*** | 0.138*** | −0.084* | 0.102* | 0.066* | −0.020 | 0.299*** | 0.187*** | −0.055* | 0.241*** | 0.162*** |
| | (0.033) | (0.055) | (0.027) | (0.029) | (0.039) | (0.029) | (0.035) | (0.039) | (0.031) | (0.028) | (0.034) | (0.022) | (0.020) | (0.046) | (0.022) |
| Far-peer GPA | 0.027 | −0.127** | −0.061* | −0.038 | −0.148** | −0.076 | −0.007 | −0.325*** | −0.193** | 0.093 | −0.097 | 0.026 | −0.147 | −0.015 | −0.041 |
| | (0.030) | (0.042) | (0.026) | (0.044) | (0.046) | (0.046) | (0.056) | (0.048) | (0.053) | (0.067) | (0.077) | (0.034) | (0.093) | (0.112) | (0.086) |
| **Race/ethnicity** | | | | | | | | | | | | | | | |
| White, non-Hispanic (reference) | | | | | | | | | | | | | | | |
| Black, non-Hispanic | 0.041* | −0.034 | −0.061*** | 0.036 | −0.002 | −0.044* | 0.024 | −0.066** | −0.056** | 0.045* | −0.018 | −0.049** | 0.014 | −0.034 | −0.030 |
| | (0.020) | (0.023) | (0.015) | (0.021) | (0.018) | (0.017) | (0.017) | (0.018) | (0.020) | (0.020) | (0.020) | (0.017) | (0.025) | (0.035) | (0.029) |
| Hispanic | 0.011 | −0.002 | −0.035 | 0.001 | 0.006 | −0.052* | 0.012 | −0.032 | −0.033 | 0.037 | −0.005 | −0.015 | 0.033 | −0.061*** | −0.037 |
| | (0.026) | (0.023) | (0.022) | (0.021) | (0.021) | (0.024) | (0.019) | (0.018) | (0.023) | (0.021) | (0.019) | (0.021) | (0.017) | (0.015) | (0.031) |
| Other race | −0.0004 | −0.009 | −0.019 | 0.004 | 0.012 | −0.004 | −0.007 | 0.003 | −0.029 | −0.004 | 0.016 | 0.006 | 0.008 | 0.009 | −0.011 |
| | (0.018) | (0.015) | (0.013) | (0.008) | (0.017) | (0.018) | (0.018) | (0.017) | (0.019) | (0.021) | (0.014) | (0.010) | (0.015) | (0.022) | (0.017) |
| **Student demographics** | | | | | | | | | | | | | | | |
| Female | 0.001 | 0.071*** | 0.0003 | 0.054*** | 0.076*** | 0.032 | 0.056* | 0.128*** | 0.010 | 0.071*** | 0.112*** | 0.016 | 0.062*** | 0.103*** | 0.057** |
| | (0.011) | (0.012) | (0.011) | (0.014) | (0.015) | (0.016) | (0.020) | (0.015) | (0.019) | (0.012) | (0.010) | (0.015) | (0.013) | (0.026) | (0.017) |
| Eligible for free or reduced priced meals | −0.003 | −0.047** | −0.027* | 0.040** | −0.043* | −0.032* | 0.030 | −0.032 | −0.035** | 0.034* | −0.028 | −0.033 | 0.039* | −0.046*** | −0.013 |
| | (0.011) | (0.014) | (0.013) | (0.011) | (0.019) | (0.011) | (0.016) | (0.022) | (0.011) | (0.012) | (0.015) | (0.017) | (0.018) | (0.012) | (0.022) |
| English language learner | 0.046** | 0.050*** | 0.036 | 0.078*** | 0.032 | 0.050* | 0.042* | 0.023 | −0.010 | 0.062*** | 0.034* | 0.015 | 0.028 | 0.040 | −0.015 |
| | (0.013) | (0.014) | (0.020) | (0.015) | (0.028) | (0.019) | (0.017) | (0.012) | (0.017) | (0.016) | (0.015) | (0.015) | (0.024) | (0.021) | (0.014) |
| Special education student | 0.033* | 0.019 | −0.002 | −0.051** | −0.004 | −0.031* | −0.031** | 0.002 | 0.004 | −0.055*** | 0.029 | 0.013 | −0.015 | −0.047*** | −0.036** |
| | (0.015) | (0.015) | (0.017) | (0.015) | (0.016) | (0.014) | (0.009) | (0.016) | (0.016) | (0.009) | (0.015) | (0.012) | (0.016) | (0.011) | (0.011) |
| Took survey in the winter term | −0.034 | −0.019 | 0.025 | −0.057** | −0.057*** | −0.029 | −0.035 | −0.099*** | −0.026 | −0.030 | −0.028 | 0.020 | −0.069* | 0.027 | 0.034 |
| | (0.021) | (0.020) | (0.018) | (0.019) | (0.015) | (0.024) | (0.018) | (0.022) | (0.017) | (0.031) | (0.025) | (0.014) | (0.027) | (0.024) | (0.029) |
| Age | 0.037*** | −0.030* | −0.008 | 0.019 | −0.042 | 0.030 | 0.073*** | −0.040* | −0.001 | 0.031* | −0.046*** | −0.024 | 0.048** | −0.059*** | −0.011 |
| | (0.010) | (0.014) | (0.012) | (0.019) | (0.023) | (0.021) | (0.017) | (0.015) | (0.024) | (0.012) | (0.009) | (0.015) | (0.013) | (0.014) | (0.016) |
| **Home language** | | | | | | | | | | | | | | | |
| English (reference) | | | | | | | | | | | | | | | |
| Spanish | 0.049* | 0.002 | −0.002 | 0.006 | 0.016 | −0.020 | −0.026 | −0.010 | −0.042 | −0.027 | 0.010 | 0.003 | 0.027 | 0.033 | −0.029 |
| | (0.019) | (0.024) | (0.018) | (0.023) | (0.021) | (0.024) | (0.020) | (0.028) | (0.030) | (0.017) | (0.019) | (0.019) | (0.025) | (0.019) | (0.031) |
| Other | 0.008 | −0.005 | 0.002 | −0.031** | −0.021 | 0.002 | −0.039 | 0.007 | 0.033 | −0.047* | 0.004 | −0.017 | −0.051* | 0.006 | −0.015 |
| | (0.013) | (0.016) | (0.015) | (0.010) | (0.016) | (0.011) | (0.020) | (0.014) | (0.020) | (0.021) | (0.012) | (0.016) | (0.020) | (0.017) | (0.013) |
| *n* | 5,903 | 5,903 | 5,903 | 4,177 | 4,177 | 4,177 | 3,606 | 3,606 | 3,606 | 5,091 | 5,091 | 5,091 | 3, 041 | 3, 041 | 3, 041 |
| $R^2$ | 0.101 | 0.095 | 0.043 | 0.117 | 0.158 | 0.075 | 0.099 | 0.163 | 0.072 | 0.080 | 0.166 | 0.067 | 0.082 | 0.136 | 0.071 |

*Notes.* Estimates calculated by running separate OLS regressions for each subgroup. Robust standard errors clustered by school. C = Conscientiousness, HWN = Hard work norm, PN = Preparedness norm.

* $p < .05$, ** $p < .01$, *** $p < .001$.

**Table S13. Subgroup analyses by eligibility for free or reduced priced meals status in Study 2**

| | Eligible for free or reduced priced meals | | | Not eligible for free or reduced priced meals | | |
|---|---|---|---|---|---|---|
| | C | HWN | PN | C | HWN | PN |
| Own GPA | 0.270*** | 0.058*** | 0.024* | 0.299*** | 0.082*** | 0.067*** |
| | (0.013) | (0.009) | (0.011) | (0.015) | (0.010) | (0.012) |
| Near-peer GPA | −0.068*** | 0.214*** | 0.123*** | −0.036* | 0.235*** | 0.151*** |
| | (0.019) | (0.025) | (0.018) | (0.016) | (0.028) | (0.017) |
| Far-peer GPA | 0.007 | −0.028 | −0.037* | 0.028 | −0.022 | 0.001 |
| | (0.016) | (0.021) | (0.017) | (0.019) | (0.026) | (0.021) |
| **Race/ethnicity** | | | | | | |
| White, non-Hispanic (reference) | | | | | | |
| Black, non-Hispanic | 0.051** | −0.042* | −0.045*** | 0.020 | −0.015 | −0.046*** |
| | (0.018) | (0.016) | (0.012) | (0.010) | (0.012) | (0.013) |
| Hispanic | 0.030 | −0.029 | −0.019 | 0.010 | −0.006 | −0.038** |
| | (0.018) | (0.016) | (0.014) | (0.013) | (0.013) | (0.011) |
| Other race | 0.008 | 0.015 | −0.007 | −0.006 | 0.001 | −0.012 |
| | (0.012) | (0.010) | (0.009) | (0.008) | (0.011) | (0.011) |
| **Grade level** | | | | | | |
| Grade 8 (reference) | | | | | | |
| Grade 9 | 0.133 | −0.029 | 0.054 | −0.141** | −0.032 | 0.075 |
| | (0.118) | (0.055) | (0.055) | (0.044) | (0.050) | (0.107) |
| Grade 10 | 0.119 | 0.025 | 0.029 | −0.141** | 0.022 | 0.064 |
| | (0.115) | (0.056) | (0.054) | (0.041) | (0.048) | (0.095) |
| Grade 11 | 0.152 | 0.082 | 0.049 | −0.160** | 0.073 | 0.081 |
| | (0.126) | (0.063) | (0.062) | (0.051) | (0.056) | (0.115) |
| Grade 12 | 0.124 | 0.075 | 0.043 | −0.144** | 0.080 | 0.059 |
| | (0.106) | (0.054) | (0.053) | (0.047) | (0.051) | (0.094) |
| **Student demographics** | | | | | | |
| Female | 0.024** | 0.093*** | 0.025* | 0.064*** | 0.100*** | 0.012 |
| | (0.008) | (0.010) | (0.011) | (0.010) | (0.009) | (0.010) |
| English language learner | 0.064*** | 0.026** | 0.030** | 0.035*** | 0.049** | 0.005 |
| | (0.011) | (0.009) | (0.010) | (0.010) | (0.015) | (0.014) |
| Special education student | −0.014 | −0.016 | −0.010 | −0.039*** | 0.030** | −0.005 |
| | (0.009) | (0.010) | (0.012) | (0.010) | (0.009) | (0.008) |
| Took survey in the winter term | −0.060*** | −0.033* | 0.012 | −0.026* | 0.001 | 0.008 |
| | (0.012) | (0.015) | (0.012) | (0.012) | (0.015) | (0.012) |
| Age | 0.102*** | −0.120*** | −0.008 | 0.102*** | −0.108*** | −0.025 |
| | (0.021) | (0.023) | (0.031) | (0.026) | (0.025) | (0.025) |
| **Home language** | | | | | | |
| English (reference) | | | | | | |
| Spanish | −0.001 | 0.020 | −0.015 | 0.018 | −0.004 | −0.017 |
| | (0.014) | (0.014) | (0.012) | (0.013) | (0.012) | (0.013) |
| Other | −0.055*** | −0.014 | −0.004 | 0.004 | 0.010 | 0.009 |
| | (0.013) | (0.010) | (0.011) | (0.010) | (0.009) | (0.010) |
| *n* | 10,704 | 10,704 | 10,704 | 11,114 | 11,114 | 11,114 |
| $R^2$ | 0.100 | 0.129 | 0.037 | 0.101 | 0.156 | 0.065 |

*Notes.* Estimates calculated by running separate OLS regressions for each subgroup. Robust standard errors clustered by school. C = Conscientiousness, HWN = Hard work norm, PN = Preparedness norm.
* p < .05, ** p < .01, *** p < .001.

**Table S14. Subgroup analyses by race/ethnicity in Study 2**

| | White | | | Black | | | Hispanic | | | Other Race | | |
|---|---|---|---|---|---|---|---|---|---|---|---|---|
| | C | HWN | PN | C | HWN | PN | C | HWN | PN | C | HWN | PN |
| Own GPA | 0.333*** | 0.076*** | 0.080*** | 0.209*** | 0.059*** | −0.003 | 0.299*** | 0.074*** | 0.049*** | 0.246*** | 0.033 | 0.057 |
| | (0.014) | (0.011) | (0.014) | (0.019) | (0.017) | (0.018) | (0.011) | (0.012) | (0.011) | (0.034) | (0.034) | (0.038) |
| Near-peer GPA | −0.001 | 0.265*** | 0.191*** | −0.071** | 0.214*** | 0.145*** | −0.064** | 0.203*** | 0.101*** | −0.086 | 0.174*** | 0.141*** |
| | (0.021) | (0.028) | (0.023) | (0.022) | (0.029) | (0.017) | (0.020) | (0.028) | (0.021) | (0.051) | (0.045) | (0.038) |
| Far-peer GPA | 0.071* | 0.029 | 0.060* | −0.030 | −0.014 | −0.028 | 0.012 | −0.058* | −0.055** | −0.037 | −0.052 | −0.025 |
| | (0.030) | (0.026) | (0.028) | (0.023) | (0.025) | (0.018) | (0.019) | (0.026) | (0.017) | (0.048) | (0.048) | (0.051) |
| **Grade level** | | | | | | | | | | | | |
| Grade 8 (reference) | | | | | | | | | | | | |
| Grade 9 | −0.019 | 0.313*** | 0.190 | −0.036 | 0.022 | −0.032 | −0.102 | −0.176*** | −0.016 | 0.263 | 0.044 | 0.298 |
| | (0.035) | (0.059) | (0.243) | (0.122) | (0.049) | (0.098) | (0.056) | (0.037) | (0.045) | (0.223) | (0.248) | (0.216) |
| Grade 10 | −0.038 | 0.324*** | 0.159 | −0.046 | 0.079 | −0.053 | −0.101 | −0.123*** | −0.031 | 0.227 | 0.123 | 0.229 |
| | (0.024) | (0.050) | (0.195) | (0.147) | (0.058) | (0.119) | (0.052) | (0.034) | (0.044) | (0.208) | (0.230) | (0.199) |
| Grade 11 | −0.066* | 0.425*** | 0.178 | −0.004 | 0.177** | −0.055 | −0.096 | −0.098* | 0.001 | 0.259 | 0.186 | 0.285 |
| | (0.029) | (0.071) | (0.245) | (0.155) | (0.064) | (0.132) | (0.061) | (0.042) | (0.050) | (0.225) | (0.266) | (0.229) |
| Grade 12 | −0.083** | 0.341*** | 0.145 | −0.019 | 0.185** | −0.022 | −0.072 | −0.078* | −0.022 | 0.227 | 0.188 | 0.215 |
| | (0.031) | (0.061) | (0.190) | (0.137) | (0.059) | (0.120) | (0.051) | (0.037) | (0.044) | (0.204) | (0.232) | (0.199) |
| **Student demographics** | | | | | | | | | | | | |
| Female | 0.073*** | 0.108*** | 0.014 | 0.048*** | 0.084*** | 0.009 | 0.025* | 0.095*** | 0.017 | 0.032 | 0.112*** | 0.074** |
| | (0.012) | (0.011) | (0.014) | (0.013) | (0.013) | (0.011) | (0.010) | (0.009) | (0.009) | (0.021) | (0.024) | (0.024) |
| Eligible for free or reduced priced meals | 0.011 | −0.037** | −0.040** | 0.021* | −0.034 | −0.029* | 0.031*** | −0.046*** | −0.016 | −0.004 | −0.023 | −0.051 |
| | (0.013) | (0.011) | (0.012) | (0.010) | (0.022) | (0.014) | (0.009) | (0.012) | (0.009) | (0.017) | (0.021) | (0.028) |
| English language learner | 0.035** | −0.003 | 0.008 | 0.025 | 0.012 | −0.011 | 0.072*** | 0.055*** | 0.029* | 0.030 | −0.019 | −0.017 |
| | (0.011) | (0.021) | (0.017) | (0.018) | (0.013) | (0.016) | (0.013) | (0.012) | (0.013) | (0.025) | (0.025) | (0.027) |
| Special education student | −0.056*** | 0.015 | −0.001 | −0.038* | 0.002 | 0.018 | 0.0001 | 0.003 | −0.025* | −0.011 | 0.021 | −0.026 |
| | (0.013) | (0.014) | (0.013) | (0.016) | (0.020) | (0.015) | (0.010) | (0.012) | (0.010) | (0.027) | (0.024) | (0.020) |
| Took survey in the winter term | 0.010 | 0.039* | 0.031 | −0.057** | −0.034 | −0.001 | −0.066*** | −0.041* | 0.001 | −0.043 | −0.025 | 0.040 |
| | (0.018) | (0.019) | (0.021) | (0.016) | (0.017) | (0.015) | (0.013) | (0.018) | (0.016) | (0.044) | (0.032) | (0.039) |
| Age | 0.142*** | −0.077 | −0.032 | 0.069* | −0.187*** | −0.012 | 0.107*** | −0.078* | −0.016 | 0.073 | −0.178** | 0.010 |
| | (0.027) | (0.044) | (0.030) | (0.026) | (0.033) | (0.051) | (0.022) | (0.031) | (0.030) | (0.047) | (0.059) | (0.060) |
| **Home language** | | | | | | | | | | | | |
| English (reference) | | | | | | | | | | | | |
| Spanish | 0.018 | 0.013 | 0.002 | −0.009 | 0.015 | −0.003 | 0.008 | 0.003 | −0.019 | 0.002 | 0.019 | 0.010 |
| | (0.010) | (0.012) | (0.014) | (0.010) | (0.015) | (0.014) | (0.013) | (0.013) | (0.011) | (0.007) | (0.026) | (0.018) |
| Other | 0.026 | 0.016 | 0.007 | −0.075*** | −0.005 | −0.002 | −0.005 | 0.003 | 0.018 | −0.030 | −0.0003 | −0.010 |
| | (0.014) | (0.012) | (0.012) | (0.016) | (0.012) | (0.018) | (0.008) | (0.010) | (0.010) | (0.027) | (0.024) | (0.019) |
| *n* | 6,166 | 6,166 | 6,166 | 5,020 | 5,020 | 5,020 | 8,913 | 8,913 | 8,913 | 1,719 | 1,719 | 1,719 |
| $R^2$ | 0.129 | 0.175 | 0.069 | 0.082 | 0.134 | 0.043 | 0.111 | 0.133 | 0.040 | 0.089 | 0.186 | 0.121 |

*Notes.* Estimates calculated by running separate OLS regressions for each subgroup. Robust standard errors clustered by school. C = Conscientiousness, HWN = Hard work norm, PN = Preparedness norm.

* $p < .05$, ** $p < .01$, *** $p < .001$.

**Table S15. Subgroup analyses by English language learner status in Study 2**

| | English Language Learner | | | Non English Language Learner | | |
|---|---|---|---|---|---|---|
| | C | HWN | PN | C | HWN | PN |
| Own GPA | 0.256*** | 0.127*** | 0.040 | 0.296*** | 0.064*** | 0.045*** |
| | (0.028) | (0.023) | (0.021) | (0.014) | (0.008) | (0.010) |
| Near-peer GPA | −0.047 | −0.034 | −0.047 | −0.058*** | 0.240*** | 0.150*** |
| | (0.035) | (0.033) | (0.035) | (0.017) | (0.023) | (0.013) |
| Far-peer GPA | 0.046 | −0.009 | −0.120** | 0.013 | −0.035 | −0.024 |
| | (0.065) | (0.079) | (0.038) | (0.016) | (0.018) | (0.013) |
| **Race/ethnicity** | | | | | | |
| White, non-Hispanic (reference) | | | | | | |
| Black, non-Hispanic | −0.042 | −0.041 | −0.111* | 0.037*** | −0.028** | −0.047*** |
| | (0.044) | (0.054) | (0.043) | (0.010) | (0.009) | (0.007) |
| Hispanic | −0.093** | 0.018 | 0.020 | 0.022* | −0.019 | −0.036*** |
| | (0.034) | (0.051) | (0.047) | (0.011) | (0.010) | (0.009) |
| Other race | −0.032 | 0.0001 | −0.013 | 0.0002 | 0.007 | −0.010 |
| | (0.022) | (0.028) | (0.027) | (0.009) | (0.008) | (0.008) |
| **Grade level** | | | | | | |
| Grade 8 (reference) | | | | | | |
| Grade 9 | 0.002 | −0.096 | −0.013 | −0.009 | −0.037 | 0.061 |
| | (0.045) | (0.049) | (0.035) | (0.094) | (0.022) | (0.078) |
| Grade 10 | −0.067** | −0.067* | −0.061 | −0.017 | 0.021 | 0.046 |
| | (0.024) | (0.033) | (0.032) | (0.089) | (0.021) | (0.072) |
| Grade 11 | | | | −0.011 | 0.077** | 0.057 |
| | (0.000) | (0.000) | (0.000) | (0.103) | (0.026) | (0.082) |
| Grade 12 | −0.031 | 0.040 | −0.022 | −0.015 | 0.076** | 0.045 |
| | (0.027) | (0.029) | (0.025) | (0.087) | (0.025) | (0.068) |
| **Student demographics** | | | | | | |
| Female | −0.033 | 0.061** | −0.047* | 0.052*** | 0.099*** | 0.026*** |
| | (0.020) | (0.021) | (0.020) | (0.007) | (0.007) | (0.007) |
| Eligible for free or reduced priced meals | 0.034 | −0.072** | 0.037 | 0.021* | −0.036*** | −0.036*** |
| | (0.017) | (0.023) | (0.020) | (0.008) | (0.010) | (0.007) |
| Special education student | 0.015 | 0.004 | −0.049 | −0.028*** | 0.009 | −0.003 |
| | (0.015) | (0.023) | (0.027) | (0.007) | (0.008) | (0.007) |
| Took survey in the winter term | −0.119*** | −0.101*** | 0.001 | −0.036** | −0.009 | 0.006 |
| | (0.022) | (0.024) | (0.024) | (0.011) | (0.013) | (0.010) |
| Age | 0.150** | −0.063 | −0.060 | 0.099*** | −0.124*** | −0.004 |
| | (0.048) | (0.045) | (0.038) | (0.018) | (0.018) | (0.022) |
| **Home language** | | | | | | |
| English (reference) | | | | | | |
| Spanish | −0.023 | −0.048 | −0.129 | 0.003 | 0.007 | −0.010 |
| | (0.079) | (0.084) | (0.079) | (0.011) | (0.009) | (0.009) |
| Other | −0.128 | −0.079 | −0.045 | −0.021 | 0.002 | 0.002 |
| | (0.091) | (0.076) | (0.074) | (0.011) | (0.006) | (0.008) |
| *n* | 2,091 | 2,091 | 2,091 | 19,727 | 19,727 | 19,727 |
| $R^2$ | 0.124 | 0.086 | 0.059 | 0.097 | 0.171 | 0.064 |

*Notes.* Estimates calculated by running separate OLS regressions for each subgroup. Robust standard errors clustered by school. C = Conscientiousness, HWN = Hard work norm, PN = Preparedness norm.
*p < .05; **p < .01; *** < .001


Benjamin Lira*, Joseph M. O'Brien*, Pablo A. Peña, Brian M. Galla, Sidney D'Mello, David S. Yeager, Amy Defnet, Tim Kautz, Kate Munkacsy, Angela L. Duckworth

**Table S16. Subgroup analyses by GPA tertiles in Study 2**

| | Low GPA | | | Medium GPA | | | High GPA | | |
|---|---|---|---|---|---|---|---|---|---|
| | C | HWN | PN | C | HWN | PN | C | HWN | PN |
| Own GPA | 0.353*** | 0.076 | 0.147*** | 0.553*** | 0.132** | 0.115*** | 0.159*** | 0.061*** | 0.036 |
| | (0.060) | (0.054) | (0.040) | (0.046) | (0.047) | (0.029) | (0.021) | (0.015) | (0.022) |
| Near-peer GPA | −0.072*** | 0.273*** | 0.145*** | −0.069** | 0.174*** | 0.120*** | −0.033 | 0.243*** | 0.149*** |
| | (0.020) | (0.027) | (0.021) | (0.021) | (0.047) | (0.022) | (0.023) | (0.027) | (0.022) |
| Far-peer GPA | 0.023 | 0.012 | −0.029 | 0.002 | −0.030 | 0.009 | 0.040 | −0.071* | −0.031 |
| | (0.022) | (0.022) | (0.022) | (0.028) | (0.031) | (0.021) | (0.028) | (0.030) | (0.019) |
| **Race/ethnicity** | | | | | | | | | |
| White, non-Hispanic (reference) | | | | | | | | | |
| Black, non-Hispanic | 0.032 | −0.005 | −0.062*** | 0.003 | −0.029 | −0.061*** | 0.083*** | −0.047** | −0.034* |
| | (0.017) | (0.015) | (0.016) | (0.019) | (0.016) | (0.016) | (0.013) | (0.015) | (0.016) |
| Hispanic | 0.033 | 0.003 | −0.058*** | 0.0001 | −0.007 | −0.003 | 0.048** | −0.033 | −0.028 |
| | (0.021) | (0.015) | (0.015) | (0.019) | (0.016) | (0.017) | (0.017) | (0.021) | (0.014) |
| Other race | 0.014 | 0.008 | −0.022* | −0.030** | 0.002 | −0.002 | 0.027 | 0.017 | −0.018 |
| | (0.017) | (0.015) | (0.010) | (0.009) | (0.010) | (0.011) | (0.016) | (0.020) | (0.018) |
| **Grade level** | | | | | | | | | |
| Grade 8 (reference) | | | | | | | | | |
| Grade 9 | −0.026 | −0.179* | −0.196*** | −0.177** | 0.024 | 0.141 | 0.249* | −0.070 | 0.316** |
| | (0.053) | (0.078) | (0.045) | (0.053) | (0.058) | (0.091) | (0.101) | (0.092) | (0.118) |
| Grade 10 | −0.052 | −0.123 | −0.184*** | −0.162** | 0.094 | 0.105 | 0.227* | −0.028 | 0.287* |
| | (0.048) | (0.073) | (0.042) | (0.050) | (0.054) | (0.084) | (0.096) | (0.088) | (0.112) |
| Grade 11 | −0.057 | −0.103 | −0.184*** | −0.148* | 0.148* | 0.107 | 0.271* | 0.039 | 0.347* |
| | (0.058) | (0.085) | (0.050) | (0.056) | (0.065) | (0.096) | (0.109) | (0.100) | (0.131) |
| Grade 12 | −0.069 | −0.082 | −0.135** | −0.120* | 0.135* | 0.073 | 0.224* | 0.049 | 0.280* |
| | (0.050) | (0.072) | (0.042) | (0.047) | (0.056) | (0.083) | (0.092) | (0.084) | (0.109) |
| **Student demographics** | | | | | | | | | |
| Female | 0.045*** | 0.085*** | 0.022* | 0.059*** | 0.112*** | 0.022 | 0.023 | 0.086*** | 0.011 |
| | (0.012) | (0.010) | (0.011) | (0.011) | (0.012) | (0.011) | (0.012) | (0.011) | (0.012) |
| Eligible for free or reduced priced meals | 0.039*** | −0.031 | −0.025 | 0.019 | −0.060*** | −0.036** | 0.021 | −0.038** | −0.027* |
| | (0.011) | (0.016) | (0.015) | (0.015) | (0.011) | (0.011) | (0.012) | (0.013) | (0.013) |
| English language learner | 0.061*** | 0.058*** | 0.033* | 0.026 | −0.005 | −0.014 | 0.057*** | 0.036* | 0.019 |
| | (0.014) | (0.012) | (0.013) | (0.017) | (0.017) | (0.019) | (0.011) | (0.014) | (0.013) |
| Special education student | −0.041** | 0.002 | −0.024* | −0.026** | 0.029** | 0.011 | −0.036*** | −0.011 | −0.014 |
| | (0.014) | (0.013) | (0.011) | (0.009) | (0.010) | (0.010) | (0.010) | (0.012) | (0.013) |
| Took survey in the winter term | −0.043* | −0.009 | −0.004 | −0.043* | 0.001 | −0.006 | −0.051*** | −0.041* | 0.031* |
| | (0.018) | (0.013) | (0.016) | (0.020) | (0.020) | (0.013) | (0.014) | (0.018) | (0.015) |
| Age | 0.155*** | −0.093** | −0.062** | 0.005 | −0.104** | 0.017 | 0.120*** | −0.132*** | −0.002 |
| | (0.032) | (0.030) | (0.023) | (0.026) | (0.034) | (0.039) | (0.034) | (0.026) | (0.037) |
| **Home language** | | | | | | | | | |
| English (reference) | | | | | | | | | |
| Spanish | 0.008 | 0.010 | 0.004 | −0.001 | −0.003 | −0.050** | 0.012 | 0.014 | −0.007 |
| | (0.018) | (0.013) | (0.015) | (0.020) | (0.015) | (0.015) | (0.016) | (0.016) | (0.016) |
| Other | −0.029* | −0.012 | −0.008 | −0.025* | 0.004 | −0.010 | −0.033* | 0.0001 | 0.016 |
| | (0.013) | (0.011) | (0.014) | (0.010) | (0.012) | (0.009) | (0.014) | (0.011) | (0.013) |
| *n* | 7,243 | 7,243 | 7,243 | 7,061 | 7,061 | 7,061 | 7,514 | 7,514 | 7,514 |
| $R^2$ | 0.061 | 0.138 | 0.062 | 0.053 | 0.155 | 0.069 | 0.071 | 0.128 | 0.038 |

*Notes.* Estimates calculated by running separate OLS regressions for each subgroup. Robust standard errors clustered by school. C = Conscientiousness, HWN = Hard work norm, PN = Preparedness norm.

* p < .05, ** p < .01, *** p < .001.

## 3. Study 3

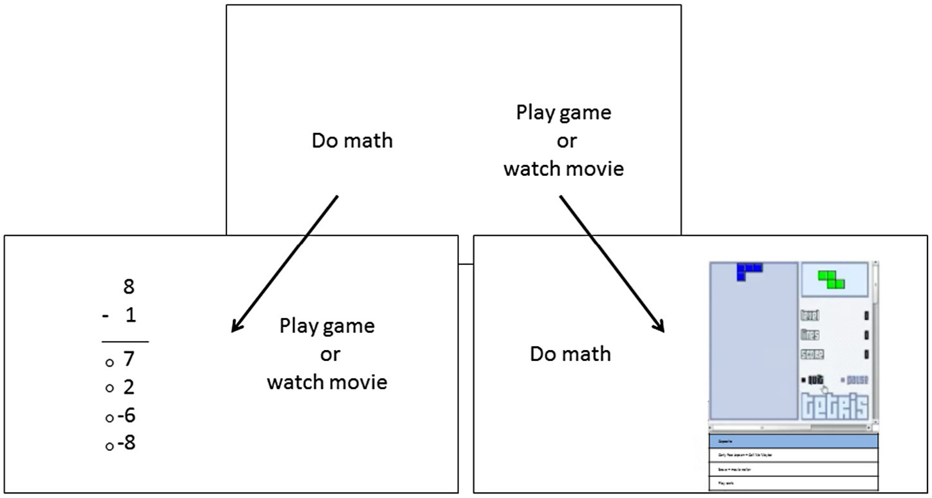


**Fig. S3.** In the Academic Diligence Task (1), students choose between “Do math” or “Play game or watch movie.” If they click “Do math,” they solve single-digit subtraction problems. If they instead click “Play game or watch movie,” a pull-down menu is displayed that contains various video clips or the option to play the video game Tetris. At any point the students can toggle between math or entertainment, but the program restricts engagement to one activity at a time. Figure reproduced with permission from Galla et al. (1).

**Table S17. Means, standard deviations, and correlation coefficients in Study 3.**

| | 1 | 2 | 3 | 4 | 5 | 6 | 7 |
|---|---|---|---|---|---|---|---|
| 1. Grit | | 0.59*** | 0.09** | 0.08** | 0.09** | 0.27*** | −0.01 |
| 2. Self-control | 0.61*** | | 0.17*** | 0.12*** | 0.03 | 0.32*** | −0.06* |
| 3. ADT | 0.06 | 0.12*** | | 0.14*** | 0.27*** | 0.25*** | 0.20*** |
| 4. College graduation | 0.03 | 0.05 | 0.18*** | | 0.19*** | 0.38*** | 0.04 |
| 5. SAT score | −0.06 | −0.12*** | 0.28*** | 0.27*** | | 0.47*** | 0.44*** |
| 6. GPA | 0.27*** | 0.31*** | 0.27*** | 0.33*** | 0.31*** | | 0.18*** |
| 7. Cognitive ability | −0.10*** | −0.16*** | 0.24*** | 0.11*** | 0.53*** | 0.16*** | |
| *M* | 3.69 | 3.50 | 10.47 | 33.49% | 921.11 | 2.91 | 63.10% |
| *SD* | 0.74 | 0.70 | 4.95 | | 184.24 | 0.67 | |
| *N* | 1,166 | 1,166 | 803 | 1,278 | 970 | 944 | 1,123 |

*Notes.* Correlations controlling for school dummy variables shown above the diagonal.
† p < .10, * p < .05, ** p < .01, *** p < .001.

**Table S18. Multilevel logistic regression models predicting college graduation using the full sample in Study 3**

| | Grit | | Self-control | | Academic Diligence Task | |
|---|---|---|---|---|---|---|
| | (1) | (2) | (1) | (2) | (1) | (2) |
| Grit: level 1 (student) | 0.186** | 0.184* | | | | |
| | (0.066) | (0.077) | | | | |
| Grit: level 2 (school) | −0.398** | −0.386** | | | | |
| | (0.125) | (0.130) | | | | |
| Missing grit | 0.155 | 0.248 | | | | |
| | (0.227) | (0.342) | | | | |
| Self-control: level 1 (student) | | | 0.278*** | 0.252** | | |
| | | | (0.068) | (0.080) | | |
| Self-control: level 2 (school) | | | −0.459*** | −0.474*** | | |
| | | | (0.121) | (0.126) | | |
| Missing self-control | | | 0.163 | 0.264 | | |
| | | | (0.227) | (0.340) | | |
| Academic Diligence Task: level 1 (student) | | | | | 0.246** | 0.213* |
| | | | | | (0.090) | (0.106) |
| Academic Diligence Task: level 2 (school) | | | | | 0.498*** | 0.413* |
| | | | | | (0.138) | (0.163) |
| Missing Academic Diligence Task | | | | | −0.134 | −0.053 |
| | | | | | (0.137) | (0.163) |
| General cognitive ability | 0.109 | 0.083 | 0.117† | 0.080 | 0.087 | 0.066 |
| | (0.071) | (0.083) | (0.071) | (0.083) | (0.071) | (0.084) |
| Missing General cognitive ability | −0.539** | −0.456* | −0.541** | −0.426† | −0.502* | −0.439† |
| | (0.207) | (0.231) | (0.208) | (0.231) | (0.208) | (0.233) |
| **Student demographics** | | | | | | |
| Age | | −0.299* | | −0.364** | | −0.292* |
| | | (0.139) | | (0.140) | | (0.139) |
| Female | | 0.613*** | | 0.540*** | | 0.596*** |
| | | (0.152) | | (0.153) | | (0.153) |
| Eligible for free or reduced-priced meals | | −0.477† | | −0.505* | | −0.461† |
| | | (0.259) | | (0.257) | | (0.261) |
| English Language Learner | | 0.388 | | 0.492 | | 0.180 |
| | | (0.330) | | (0.322) | | (0.345) |
| Special Education Student | | 0.362 | | 0.359 | | 0.483 |
| | | (0.371) | | (0.371) | | (0.372) |
| **Race/ethnicity** | | | | | | |
| White | | 0.145 | | 0.087 | | 0.203 |
| | | (0.475) | | (0.479) | | (0.481) |
| Hispanic | | −0.023 | | −0.142 | | 0.080 |
| | | (0.222) | | (0.228) | | (0.222) |
| Asian | | 0.187 | | 0.136 | | 0.212 |
| | | (0.344) | | (0.341) | | (0.346) |
| Other | | −0.401 | | −0.398 | | −0.358 |
| | | (0.370) | | (0.372) | | (0.370) |
| Constant | −0.731*** | 4.768† | −0.730*** | 6.036* | −0.637*** | 4.645† |
| | (0.125) | (2.491) | (0.117) | (2.512) | (0.132) | (2.492) |
| Observations | 1,278 | 937 | 1,278 | 937 | 1,278 | 937 |
| Log Likelihood | -775.303 | -566.788 | -769.586 | -563.423 | -775.282 | -569.294 |
| Akaike Inf. Crit. | 1,564.605 | 1,165.576 | 1,553.171 | 1,158.846 | 1,564.563 | 1,170.589 |
| Bayesian Inf. Crit. | 1,600.677 | 1,243.059 | 1,589.242 | 1,236.329 | 1,600.634 | 1,248.072 |
| AUC | 0.671 | 0.696 | 0.678 | 0.692 | 0.674 | 0.695 |

*Notes.* For each variable we report 2 models. (1) Unadjusted, (2) Controlling for student characteristics. Level 2 is the empirical bayesian estimate of the school mean. Level 1 is the deviation of each individual relative to the estimated school mean. Numeric variables were standardized prior to estimation, so coefficients are betas. Categorical variables are not standardized.
† < p .10, * p < .05, ** p < .01, *** p < .001.

**Table S19. Models Predicting College Graduation from Self-Reported Grit and Self-Control, and the Academic Diligence Task (untransformed)**

| | Grit | | Self-control | | Academic Diligence Task | |
|---|---|---|---|---|---|---|
| | (1) | (2) | (1) | (2) | (1) | (2) |
| Grit: level 1 (student) | 0.186** | 0.184* | | | | |
| | (0.066) | (0.077) | | | | |
| Grit: level 2 (school) | −0.398** | −0.386** | | | | |
| | (0.125) | (0.130) | | | | |
| Missing grit | 0.155 | 0.248 | | | | |
| | (0.227) | (0.342) | | | | |
| Self-control: level 1 (student) | | | 0.278*** | 0.252** | | |
| | | | (0.068) | (0.080) | | |
| Self-control: level 2 (school) | | | −0.459*** | −0.474*** | | |
| | | | (0.121) | (0.126) | | |
| Missing self-control | | | 0.163 | 0.264 | | |
| | | | (0.227) | (0.340) | | |
| Academic Diligence Task: level 1 (student) | | | | | −0.103 | −0.157 |
| | | | | | (0.127) | (0.148) |
| Academic Diligence Task: level 2 (school) | | | | | 0.318* | 0.182 |
| | | | | | (0.154) | (0.176) |
| Missing Academic Diligence Task | | | | | −0.112 | −0.011 |
| | | | | | (0.136) | (0.162) |
| General cognitive ability | 0.109 | 0.083 | 0.117† | 0.080 | 0.113 | 0.088 |
| | (0.071) | (0.083) | (0.071) | (0.083) | (0.070) | (0.083) |
| Missing General cognitive ability | −0.539** | −0.456* | −0.541** | −0.426† | −0.511* | −0.447† |
| | (0.207) | (0.231) | (0.208) | (0.231) | (0.208) | (0.232) |
| **Student demographics** | | | | | | |
| Age | | −0.299* | | −0.364** | | −0.295* |
| | | (0.139) | | (0.140) | | (0.138) |
| Female | | 0.613*** | | 0.540*** | | 0.633*** |
| | | (0.152) | | (0.153) | | (0.152) |
| Eligible for free or reduced-priced meals | | −0.477† | | −0.505* | | −0.392 |
| | | (0.259) | | (0.257) | | (0.262) |
| English Language Learner | | 0.388 | | 0.492 | | 0.228 |
| | | (0.330) | | (0.322) | | (0.342) |
| Special Education Student | | 0.362 | | 0.359 | | 0.459 |
| | | (0.371) | | (0.371) | | (0.371) |
| **Race/ethnicity** | | | | | | |
| White | | 0.145 | | 0.087 | | 0.197 |
| | | (0.475) | | (0.479) | | (0.480) |
| Hispanic | | −0.023 | | −0.142 | | 0.151 |
| | | (0.222) | | (0.228) | | (0.220) |
| Asian | | 0.187 | | 0.136 | | 0.240 |
| | | (0.344) | | (0.341) | | (0.345) |
| Other | | −0.401 | | −0.398 | | −0.332 |
| | | (0.370) | | (0.372) | | (0.368) |
| Constant | −0.731*** | 4.768† | −0.730*** | 6.036* | −0.638*** | 4.568† |
| | (0.125) | (2.491) | (0.117) | (2.512) | (0.129) | (2.480) |
| Observations | 1,278 | 937 | 1,278 | 937 | 1,278 | 937 |
| Log Likelihood | -775.303 | -566.788 | -769.586 | -563.423 | -778.971 | -571.037 |
| Akaike Inf. Crit. | 1,564.605 | 1,165.576 | 1,553.171 | 1,158.846 | 1,571.943 | 1,174.075 |
| Bayesian Inf. Crit. | 1,600.677 | 1,243.059 | 1,589.242 | 1,236.329 | 1,608.014 | 1,251.557 |
| AUC | 0.671 | 0.696 | 0.678 | 0.692 | 0.674 | 0.695 |

*Notes.* For each variable we report 2 models. (1) Unadjusted, (2) Controlling for student characteristics. Level 2 is the empirical bayesian estimate of the school mean. Level 1 is the deviation of each individual relative to the estimated school mean. Numeric variables were standardized prior to estimation, so coefficients are betas. Categorical variables are not standardized.
† < p .10, * p < .05, ** p < .01, *** p < .001.

**Table S20. Multilevel logistic regression models predicting college graduation using listwise deletion in Study 3**

| | Grit | | Self-control | | Academic Diligence Task | |
|---|---|---|---|---|---|---|
| | (1) | (2) | (1) | (2) | (1) | (2) |
| Grit: level 1 (student) | 0.163* | 0.151† | | | | |
| | (0.070) | (0.083) | | | | |
| Grit: level 2 (school) | −0.385** | −0.343* | | | | |
| | (0.135) | (0.136) | | | | |
| Self-control: level 1 (student) | | | 0.285*** | 0.226** | | |
| | | | (0.072) | (0.085) | | |
| Self-control: level 2 (school) | | | −0.460*** | −0.448*** | | |
| | | | (0.130) | (0.118) | | |
| Academic Diligence Task: level 1 (student) | | | | | 0.334** | 0.280* |
| | | | | | (0.106) | (0.128) |
| Academic Diligence Task: level 2 (school) | | | | | 0.682*** | 0.553** |
| | | | | | (0.164) | (0.195) |
| General cognitive ability | 0.080 | 0.064 | 0.091 | 0.064 | 0.008 | 0.025 |
| | (0.074) | (0.085) | (0.074) | (0.085) | (0.089) | (0.105) |
| **Student demographics** | | | | | | |
| Age | | −0.320* | | −0.366* | | −0.268 |
| | | (0.151) | | (0.149) | | (0.183) |
| Female | | 0.559*** | | 0.477** | | 0.527** |
| | | (0.165) | | (0.167) | | (0.200) |
| Eligible for free or reduced-priced meals | | −0.703* | | −0.788** | | −0.661† |
| | | (0.300) | | (0.305) | | (0.343) |
| English Language Learner | | 0.417 | | 0.533† | | 0.306 |
| | | (0.370) | | (0.322) | | (0.472) |
| Special Education Student | | 0.079 | | 0.074 | | 0.459 |
| | | (0.446) | | (0.446) | | (0.661) |
| **Race/ethnicity** | | | | | | |
| White | | −0.060 | | −0.108 | | −0.097 |
| | | (0.514) | | (0.513) | | (0.553) |
| Hispanic | | 0.015 | | −0.115 | | 0.115 |
| | | (0.249) | | (0.244) | | (0.276) |
| Asian | | 0.196 | | 0.172 | | 0.216 |
| | | (0.375) | | (0.370) | | (0.430) |
| Other | | −0.539 | | −0.519 | | −0.241 |
| | | (0.405) | | (0.408) | | (0.475) |
| Constant | −0.750*** | 5.391* | −0.751*** | 6.377* | −0.655*** | 4.385 |
| | (0.133) | (2.711) | (0.124) | (2.680) | (0.137) | (3.280) |
| Observations | 1,037 | 780 | 1,037 | 780 | 740 | 552 |
| Log Likelihood | -639.542 | -478.316 | -633.036 | -474.456 | -457.054 | -339.973 |
| Akaike Inf. Crit. | 1,289.083 | 984.633 | 1,276.072 | 976.913 | 924.108 | 707.947 |
| Bayesian Inf. Crit. | 1,313.804 | 1,049.863 | 1,300.793 | 1,042.143 | 947.141 | 768.337 |
| AUC | 0.671 | 0.696 | 0.678 | 0.692 | 0.674 | 0.695 |

*Notes.* For each variable we report 2 models. (1) Unadjusted, (2) Controlling for student characteristics. Level 2 is the empirical bayesian estimate of the school mean. Level 1 is the deviation of each individual relative to the estimated school mean. Numeric variables were standardized prior to estimation, so coefficients are betas. Categorical variables are not standardized.
† < p .10, * p < .05, ** p < .01, *** p < .001.

**Table S21. OLS regression models predicting self-reported grit and self-control, and the Academic Diligence Task from peer and own performance in Study 3 (conceptual replication of Studies 1 and 2).**

| | ADT | | | | Grit | | | | Self-Control | | | |
|---|---|---|---|---|---|---|---|---|---|---|---|---|
| | CG | CA | GPA | SAT | CG | CA | GPA | SAT | CG | CA | GPA | SAT |
| Own performance | 0.117 | 0.204** | 0.182** | 0.286*** | 0.073 | −0.018 | 0.315*** | 0.079 | 0.095*** | −0.060 | 0.328*** | 0.027 |
| | (0.058) | (0.046) | (0.072) | (0.073) | (0.058) | (0.037) | (0.064) | (0.075) | (0.018) | (0.037) | (0.013) | (0.048) |
| Peer performance | 0.016 | 0.078** | 0.023 | −0.110 | −0.212*** | −0.214*** | −0.005 | −0.260*** | −0.239*** | −0.243*** | −0.066*** | −0.230*** |
| | (0.053) | (0.024) | (0.072) | (0.08) | (0.041) | (0.04) | (0.038) | (0.054) | (0.028) | (0.047) | (0.017) | (0.021) |
| **Student demographics** | | | | | | | | | | | | |
| Eligible for free or reduced-price meals | −0.026 | 0.064 | −0.055 | 0.010 | 0.050 | 0.099 | 0.258* | 0.109 | 0.053 | 0.033 | 0.305 | 0.054 |
| | (0.152) | (0.237) | (0.116) | (0.157) | (0.065) | (0.141) | (0.104) | (0.151) | (0.184) | (0.09) | (0.155) | (0.585) |
| Female | 0.096 | 0.138 | 0.032 | 0.145 | −0.048 | −0.013 | −0.114 | −0.007 | 0.200*** | 0.268*** | 0.097 | 0.270*** |
| | (0.117) | (0.116) | (0.16) | (0.083) | (0.047) | (0.065) | (0.055) | (0.088) | (0.034) | (0.045) | (0.051) | (0.043) |
| English language learner | 0.338 | 0.379* | 0.373 | 0.062 | −0.041 | −0.229 | −0.297 | −0.689* | 0.264** | 0.074 | 0.051 | −0.261 |
| | (0.189) | (0.159) | (0.32) | (0.663) | (0.321) | (0.242) | (0.223) | (0.309) | (0.092) | (0.081) | (0.082) | (0.134) |
| Special education student | −0.491* | −0.490* | −0.220 | −0.079 | 0.221 | 0.325*** | 0.431 | 0.296** | 0.226 | 0.172 | 0.486*** | 0.161 |
| | (0.222) | (0.21) | (0.227) | (0.305) | (0.118) | (0.091) | (0.237) | (0.077) | (0.106) | (0.165) | (0.135) | (0.148) |
| **Race/ethnicity** | | | | | | | | | | | | |
| White | 0.198 | 0.177 | 0.153 | 0.129 | 0.087 | 0.079 | −0.046 | 0.074 | −0.038 | −0.022 | −0.019 | −0.139 |
| | (0.133) | (0.183) | (0.091) | (0.191) | (0.225) | (0.334) | (0.077) | (0.28) | (0.312) | (0.431) | (0.131) | (0.787) |
| Hispanic | 0.229* | 0.011 | 0.246 | 0.203* | −0.071 | 0.047 | −0.083 | −0.066 | −0.190 | −0.071 | −0.267** | −0.191*** |
| | (0.097) | (0.099) | (0.14) | (0.085) | (0.164) | (0.097) | (0.14) | (0.11) | (0.121) | (0.069) | (0.083) | (0.039) |
| Asian | 0.645*** | 0.532*** | 0.604*** | 0.519*** | 0.194 | 0.227 | −0.121 | 0.177 | 0.077 | 0.149 | −0.220 | −0.214 |
| | (0.117) | (0.089) | (0.09) | (0.118) | (0.446) | (0.22) | (0.365) | (0.114) | (0.123) | (0.182) | (0.143) | (0.158) |
| Other | 0.054 | 0.009 | 0.145 | 0.031 | 0.161 | 0.133 | 0.054 | 0.209 | −0.099 | −0.174 | −0.188 | −0.216 |
| | (0.224) | (0.2) | (0.287) | (0.22) | (0.192) | (0.195) | (0.253) | (0.227) | (0.166) | (0.216) | (0.188) | (0.222) |
| Intercept | −0.218 | −0.211 | −0.211 | −0.293 | 0.000 | −0.134 | −0.035 | −0.074 | −0.112 | −0.195 | −0.155 | −0.149 |
| | (0.332) | (0.393) | (0.308) | (0.23) | (0.084) | (0.162) | (0.105) | (0.189) | (0.174) | (0.099) | (0.152) | (0.462) |

*Notes.* ADT = Academic Diligence Task, CG = College graduation, CA = Cognitive ability. Standard errors were calculated from the 95% bootstrap confidence intervals. They are calculated under the assumption of symmetric confidence intervals that are t-distributed. These assumptions do not necessarily hold in the wild bootstrap procedure.

† $p < .10$, * $p < .05$, ** $p < .01$, *** $p < .001$.

**SI Dataset S1 (S2-CleanData.dta)**
Click here for dataset for Study 2.

**SI Dataset S2 (S3-CleanData.rda)**
Click here for dataset for Study 3.

## References


1. BM Galla, et al., The Academic Diligence Task (ADT): Assessing individual differences in effort on tedious but important schoolwork. *Contemp. Educ. Psychol.* **39**, 314–325 (2014).
2. CC Clogg, E Petkova, A Haritou, Statistical Methods for Comparing Regression Coefficients Between Models. *Am. J. Sociol.* **100**, 1261–1293 (1995).
3. Y Benjamini, D Yekutieli, The control of the false discovery rate in multiple testing under dependency. *The Annals Stat.* **29** (2001).
4. E Tsukayama, AL Duckworth, B Kim, Domain-specific impulsivity in school-age children. *Dev. Sci.* **16**, 879–893 (2013).
5. AL Duckworth, ME Seligman, Self-Discipline Outdoes IQ in Predicting Academic Performance of Adolescents. *Psychol. Sci.* **16**, 939–944 (2005).
